# Drug Delivery (Nano)Platforms for Oral and Dental Applications: Tissue Regeneration, Infection Control, and Cancer Management

**DOI:** 10.1002/advs.202004014

**Published:** 2021-02-05

**Authors:** Pooyan Makvandi, Uros Josic, Masoud Delfi, Filippo Pinelli, Vahid Jahed, Emine Kaya, Milad Ashrafizadeh, Atefeh Zarepour, Filippo Rossi, Ali Zarrabi, Tarun Agarwal, Ehsan Nazarzadeh Zare, Matineh Ghomi, Tapas Kumar Maiti, Lorenzo Breschi, Franklin R Tay

**Affiliations:** ^1^ Chemistry Department, Faculty of Science Shahid Chamran University of Ahvaz Ahvaz 6153753843 Iran; ^2^ Department of Biomedical and Neuromotor Sciences University of Bologna Via San Vitale 59 Bologna 40125 Italy; ^3^ Department of Chemical Sciences University of Naples “Federico II” Complesso Universitario Monte S. Angelo, Via Cintia Naples 80126 Italy; ^4^ Department of Chemistry, Materials and Chemical Engineering Politecnico di Milano Technical University Milano 20133 Italy; ^5^ Biomedical Engineering Division, Faculty of Chemical Engineering Tarbiat Modares University Tehran Iran; ^6^ Faculty of Dentistry Istanbul Okan University Tuzla Campus Tuzla Istanbul 34959 Turkey; ^7^ Faculty of Engineering and Natural Sciences Sabanci University Orta Mahalle, Üniversite Caddesi No. 27, Orhanlı Tuzla Istanbul 34956 Turkey; ^8^ Sabanci University Nanotechnology Research and Application Center (SUNUM) Tuzla Istanbul 34956 Turkey; ^9^ Department of Biotechnology Indian Institute of Technology Kharagpur Kharagpur West Bengal 721302 India; ^10^ School of Chemistry Damghan University Damghan 36716–41167 Iran; ^11^ The Dental College of Georgia Augusta University 1430 John Wesley Gilbert Drive Augusta GA 30192 USA; ^12^ The Graduate School Augusta University Augusta GA 30912 USA

**Keywords:** antibacterial, antiviral, drug delivery, oral cancer, tissue regeneration

## Abstract

The oral cavity and oropharynx are complex environments that are susceptible to physical, chemical, and microbiological insults. They are also common sites for pathological and cancerous changes. The effectiveness of conventional locally‐administered medications against diseases affecting these oral milieus may be compromised by constant salivary flow. For systemically‐administered medications, drug resistance and adverse side‐effects are issues that need to be resolved. New strategies for drug delivery have been investigated over the last decade to overcome these obstacles. Synthesis of nanoparticle‐containing agents that promote healing represents a quantum leap in ensuring safe, efficient drug delivery to the affected tissues. Micro/nanoencapsulants with unique structures and properties function as more favorable drug‐release platforms than conventional treatment approaches. The present review provides an overview of newly‐developed nanocarriers and discusses their potential applications and limitations in various fields of dentistry and oral medicine.

## Graphical abstract

A wide variety of micro/nanoscale platforms are employed for oral and dental applications including tissue regeneration, infection control, and cancer management. Such micro and nanocarriers deliver ions (e.g., fluoride, calcium, strontium), antibiotic, antiviral, antifungal compounds, as well as genes and proteins.

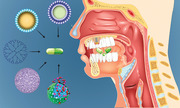

## 1 Introduction

**Figure 1:**
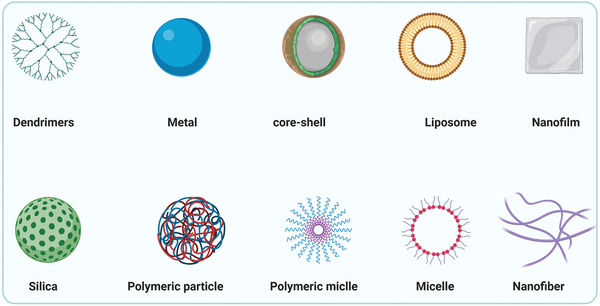
Schematic of nanoscopical drug delivery systems employed in dentistry and for treatment of oral cancer.

For a long time, systemic administration has been the most important route for delivery of drugs for treating oral diseases. This pathway of drug administration produces problems such as drug resistance and dysbiosis, as well as adverse side effects on tissues and organs unrelated to the targeted tissue. Because of these catches, much effort has been devoted to the development of more effective localized drug delivery strategies.^[^ ^1^ ^]^ The most significant advantages of using drug carriers are the possibility to control and target drug release, improve pharmacokinetics, increase drug bioavailability and selectivity, and thence, treatment effectiveness.^[^ ^2^, ^3^ ^]^ The use of drug carriers also improves the safety of administration and limits the interaction of drugs with other body tissues. Although different drug carriers are available for the treatment of diseases,^[^ ^1^, ^4^, ^5^ ^]^ there is an ongoing tendency to use nanoscopical drug delivery systems for dental applications (**Figure** **1**).^[^ ^6^, ^7^ ^]^ Nanoparticles are one of the important nanodevices used as drug delivery systems. They have different structures such as nanospheres, nanocapsules, core‐shell nanoparticles, mesoporous nanoparticles, just to mention a few.^[^ ^8^ ^]^

In the present review, an impressive array of bioactive compounds that can be delivered by micro/nanocarriers such as antibiotics, antifungals, ions, genes, and proteins will be presented in depth. Drug delivery platforms for oral applications such as tissue regeneration, infection control, and cancer management will also be discussed.

## 2 Antibiotic Delivery

**Figure 2:**
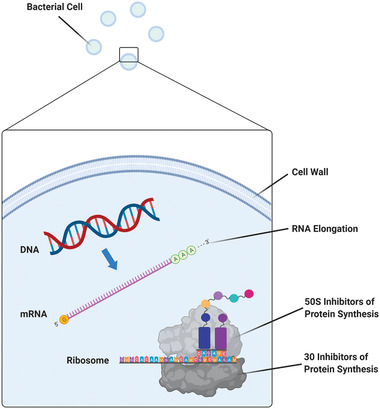
The antibacterial mechanisms of antibiotics are broadly classified into: inhibition or regulation of enzymes involved in cell wall synthesis, interference with nucleic acid metabolism, inhibition of protein synthesis, and disruption of bacterial cell membrane structure. Reproduced with permission.^[^ ^16^ ^]^ Copyright 2017, CMB Association.

Eradication of microbial biofilms is a major area of concern in the field of oral health. Bacterial biofilms are derived from a consortium of bacterial clusters that produce an extracellular matrix known as extracellular polymeric substance (EPS) around individual cells. The EPS consists of different proteins and polysaccharides which provide binding sites. The microenvironment created by the EPS enables the bacteria to stick to one another and adhere to biological or non‐biological surfaces. Drug tolerance of dental biofilms increases when they mature. Some bacteria such as *Streptococcus mutans* and *lactobacilli* also create an acidic environment that reduces the effectiveness of antibiotics.^[^ ^9^, ^10^ ^]^ To solve these problems, new drug delivery platforms have emerged for preventing and treatment of oral infectious diseases.^[^ ^11^, ^12^ ^]^ The administration of these platforms improves drug‐pathogen interactions and eliminates drug depletion and drug overuse.^[^ ^11^, ^13^, ^14^, ^15^ ^]^ Regardless of whether antibiotics are administered systemically or locally, their mechanisms of action remain the same and are illustrated in **Figure** **2**.

Over the last couple of decades, there has been an upsurge of interest in using microscopical and nanoscopical materials for dental applications. Whereas active antibiotics are mixed with dental materials in conventional delivery methods, antimicrobial agents are immobilized on the micro‐ and nanomaterials for improved stability, localized delivery, and sustained release.^[^ ^17^ ^]^

**Figure 3:**
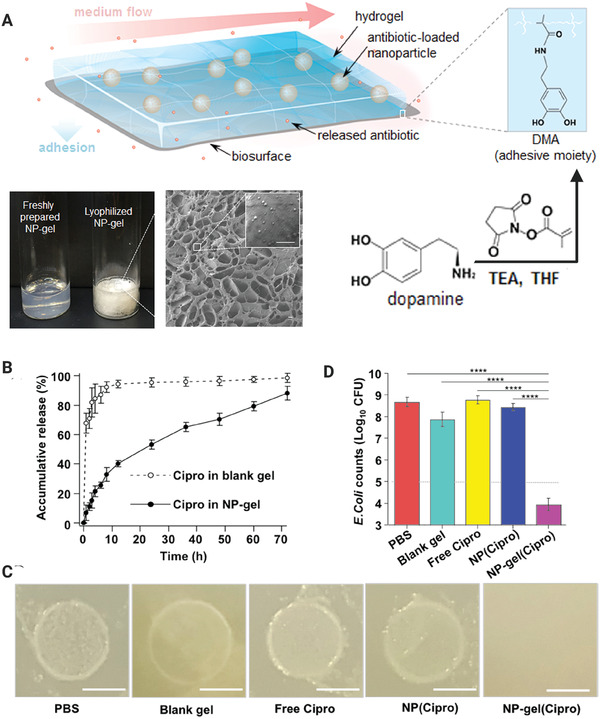
A) Top and bottom right: Schematic of an adhesive nanoparticle‐hydrogel (NP‐gel) hybrid system for localized antibiotic release to inhibit bacterial growth under flow conditions. Bottom left: Photographs of freshly prepared and lyophilized samples; a scanning electron microscope image of the lyophilized sample is included (bar = 1 µm). B) The release profile of ciprofloxacin (Cipro) from NP‐gel, in which Cipro was loaded into the embedded nanoparticles (left panel). Quantification of bacterial load of the *Escherichia coli* biofilm samples (right panel). C) Photographs of *E. coli* biofilms after treatment with phosphate‐buffered saline (PBS), blank gel (without nanoparticles or Cipro), free Cipro, Cipro‐loaded nanoparticles ((NP(Cipro), without hydrogel), and Cipro‐loaded NP−gel (NP−gel(Cipro)). Reproduced with permission.^[^ ^18^ ^]^ Copyright 2016, American Chemical Society.

The oral cavity is a complex environment in which shear forces are constantly present as a consequence of the action of the tongue against the palate and the oral mucosa against the teeth. The shear rate depends on the viscosity of the bolus and the level of lubrication.^[^ ^19^ ^]^ Changes in shear stress can influence the characteristics of oral biofilms, such as their morphology, thickness, and diversity.^[^ ^20^ ^]^ This, bacterial infections in areas with high shear forces are challenging to be managed and difficult to be eradicated. A novel antimicrobial strategy that takes advantage of the existing shear forces in living organisms has been proposed and investigated. **Figure** **3** shows the mechanism of action of a nanoparticle‐hydrogel hybrid system and its effective release of antibiotics under shear stresses. Although tested on mouse skin, this drug delivery method represents a potential strategy for oral application because it did not introduce any toxic effect on the tissue and was able to prevent bacterial biofilm formation.^[^ ^18^ ^]^

Infections have always been challenging in the surgical placement of orthopedic and dental implants. One of the possible reasons for dental implant failure is post‐surgical osteomyelitis, which invariably necessitates implant removal. Osteomyelitis, the infection of the bone, is caused mostly by three types of bacteria, *Staphylococcus*, *Enterobacteriacea*, and *Pseudomonas*, in which *S. aureus* and *S. epidermis* are the main pathogens.^[^ ^21^, ^22^, ^23^ ^]^ The majority of implant‐related infections are caused by bacterial adhesion, although biofilm formation at the implantation sites may also trigger infection. One of the most effective steps in preventing implant‐related infections is the inhibition of bacterial adhesion.^[^ ^24^ ^]^ Conventional treatment of osteomyelitis involves antibiotic therapy and debridement of the infected tissues.^[^ ^22^, ^25^, ^26^ ^]^ However, systemic antibiotic delivery is associated with increasing renal and liver toxicity due to ineffective penetration of the antibiotics into the cells and excessive antibiotic intake.^[^ ^24^, ^27^ ^]^ Thus, targeted release of antibiotics directly to the infected tissues is more desirable than conventional treatment.

Treatment of osteomyelitis with targeted antibacterial delivery is a novel therapeutic method that increases the amount of antibiotics delivered to the infected sites without causing systemic toxicity. Drug‐loaded calcium phosphate‐based coatings (e.g., hydroxyapatite) have been experimentally investigated for the treatment of osteomyelitis. Hydroxyapatite is biocompatible, non‐toxic, non‐immunogenic, and possesses relatively potent antibacterial activity as well as bioactivity toward bone regeneration.^[^ ^28^, ^29^, ^30^ ^]^ Hydroxyapatite has high adsorption capacity because its positively‐charged surface interacts with deprotonated carboxyl groups and other negatively‐charged groups present in the antibiotic molecules.^[^ ^31^ ^]^

**Figure 4:**
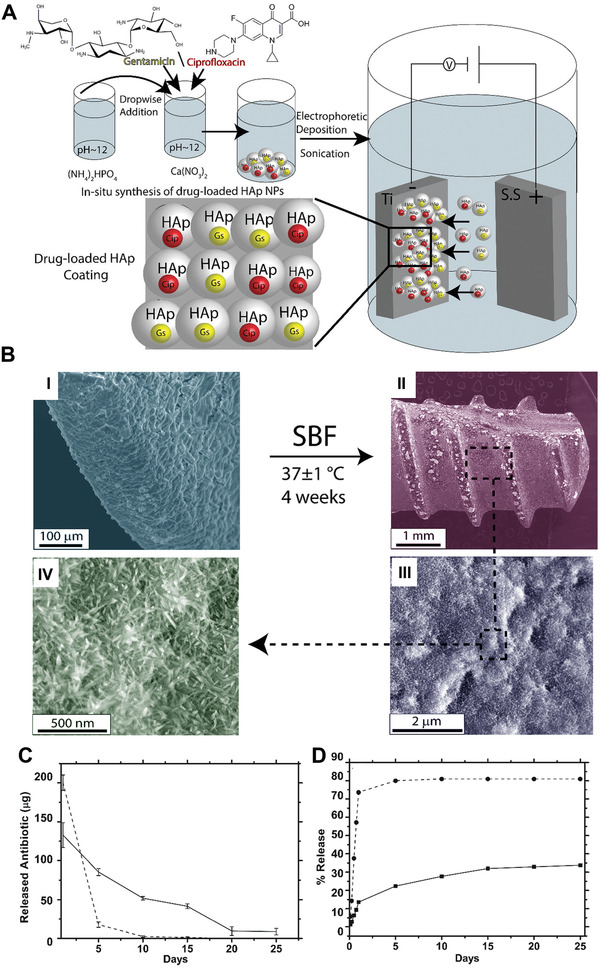
A) Schematic of the synthesis and deposition of antibiotics‐loaded hydroxyapatite (HAp) nanoparticles. B) Scanning electron microscopy images of a dental implant surface coated with gentamicin‐HAp nanoparticles I) before and II–IV) after 4 weeks of immersion in simulated body fluid at 37 °C. C,D) The release profile of gentamicin sulfate‐loaded HAp (solid lines) and ciprofloxacin‐loaded HAp (dashed lines). C) Total amount of antibiotic release. D) Cumulative release percentages. Reproduced with permission.^[^ ^32^ ^]^ Copyright 2017, Royal Society of Chemistry.

Hydroxyapatite coatings loaded with antibiotics are used as bone implant coatings to prevent bacterial adhesion. A technique was used to coat titanium implants by a single‐stage electrophoretically‐driven deposition of hydroxyapatite nanoparticles loaded with antibiotics (**Figure** **4**A). In this approach, pristine hydroxyapatite nanoparticles are loaded with gentamicin sulfate or ciprofloxacin, followed by single‐step electrophoretic deposition to create osteoconductive and antibacterial‐coated nanoparticles that are capable of sustained release of the two antibiotics. A bioactivity study was performed on a commercially available dental titanium implant coated with gentamicin sulfate‐hydroxyapatite using scanning electron microscopy (Figure 4B). According to the microscopy images, the nanoparticle coating covered the titanium implant homogenously. Microscopy images of the coated implants after 4 weeks of immersion in simulated body fluid at 37 °C showed excellent retention of the hydroxyapatite coating by the titanium implant (Figure 4B). Examination of the kinetics of antibiotic release as a function of time (Figure 4C,D) indicated a burst release profile for the two antibiotics during the first day and a slower sustained release profile that continued for 10 days for ciprofloxacin and 25 days for gentamicin sulfate.^[^ ^32^ ^]^

## 3 Antifungal Delivery

Human involvement with fungal diseases has escalated exponentially in recent years. Fungal infections can cause mortality in case of neglect, particularly for habitants in tropical countries where the weather plays a key role in fungal growth. The oral cavity, the genital organs, skin, hair, and nails are the most common sites for superficial fungal infections.^[^ ^33^, ^34^ ^]^

Oral fungal infections include candidiasis, mucormycosis, histoplasmosis, blastomycosis, aspergillosis, cryptococcosis, geotrichosis, and coccidioidomycosis. Among these, the most common oral fungal infection is candidiasis, which is also the least hazardous. Candida infections may be identified by the appearance of a white cottage cheese‐like film (i.e., thrush) in the oral cavity.^[^ ^35^ ^]^ Hyperplastic candidiasis, known previously as candida leukoplakia, is the most widespread variant of candidiasis caused mostly by *Candida albicans*, and is manifested by the appearance of white patches on the commissures of the oral mucosa; if the lesions are untreated, a small portion of those lesions may undergo dysplasia and develop carcinoma.^[^ ^36^ ^]^ The second most common fungal infection is aspergillosis caused by Aspergillus species such as *A. fumigatus* and *A. flavus*.^[^ ^37^ ^]^ Deaths from invasive aspergillosis have significantly increased.^[^ ^38^ ^]^ This type of fungal infection may spread from the primary oral mucosa infection site to the maxillary sinus.^[^ ^39^ ^]^

**Figure 5:**
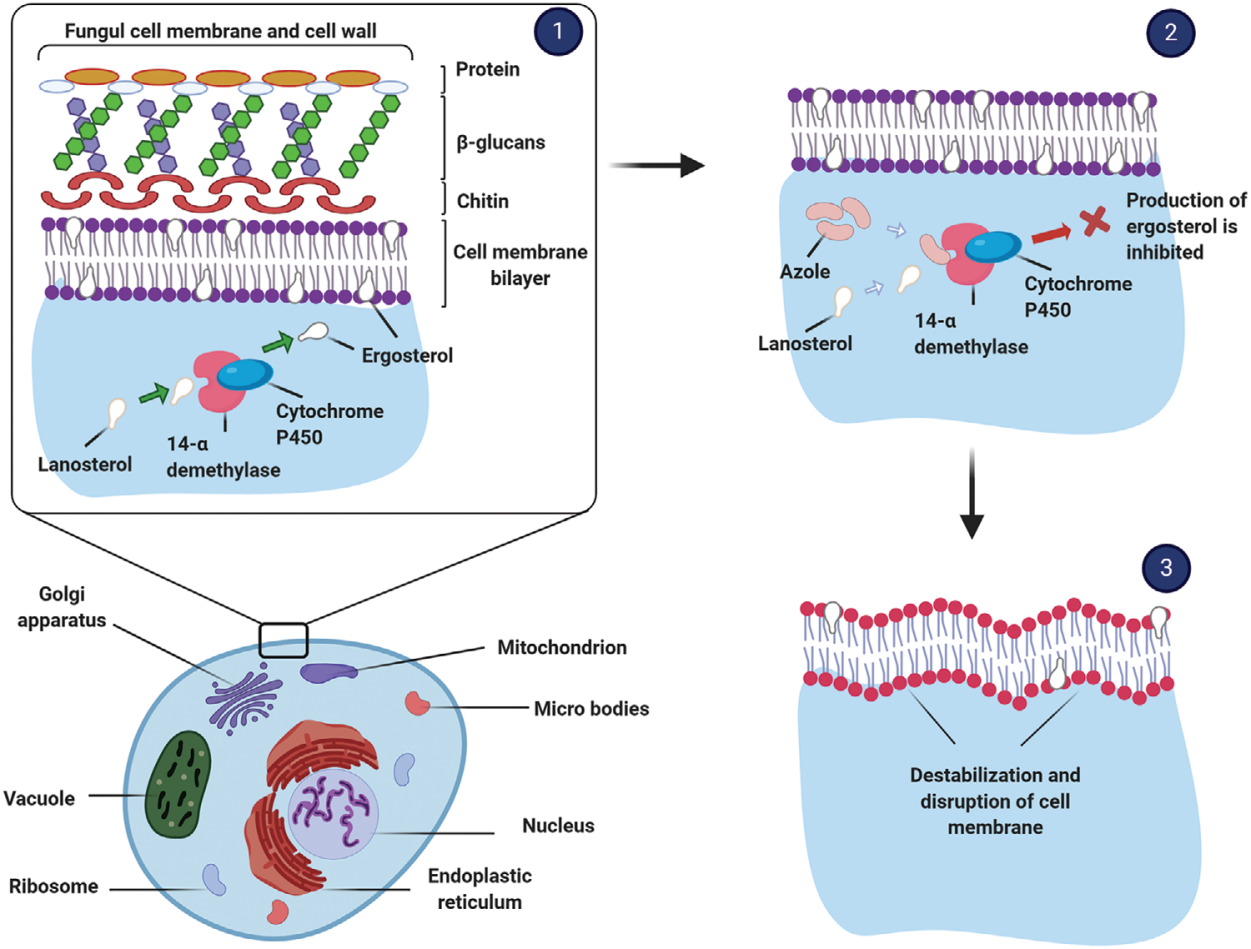
The mechanism of action of azole group antifungal agents: 1) inhibition of the conversion of lanosterol to ergosterol, 2) inhibition of ergosterol production, and 3) destabilization and disruption of the fungal cell membrane.

Different antifungal drugs have been used to combat the aforementioned infection, the best‐known examples being the azole class of drugs such as clotrimazole, miconazole, econazole, oxiconazole, tioconazole, and sertaconazole. The mechanism of action of azole antifungals is summarized in **Figure** **5**. Azoles inhibit C‐14 *α*‐demethylase (a cytochrome P450 enzyme) and block the demethylation of lanosterol to ergosterol, the principal sterol of fungal membranes. Disruption of the fungal membrane structure and function inhibits fungal cell growth. However, the hydrophobic nature of these azole antifungal drugs render them sparingly water‐soluble, resulting in poor oral bioavailability.^[^ ^40^, ^41^, ^42^ ^]^ There are two additional drawbacks that hinder the clinical applications of these antifungal agents: toxicity and drug‐drug interactions.^[^ ^43^ ^]^ For example, although amphotericin‐B is a potent antifungal drug, its use may cause infusion‐related reactions and nephrotoxicity. In addition, the simultaneous use of amphotericin‐B with other drugs such as cyclosporine and aminoglycosides increases its nephrotoxicity.^[^ ^44^ ^]^ This example illustrates the need for the design and creation of efficient drug delivery platforms.

Some of the obstacles of antifungal drug delivery have been overcome by the introduction of drug delivery nanoparticles. For example, lipid‐based formulations of amphotericin‐B consisting of amphotericin‐B lipid complex, amphotericin‐B colloidal dispersion, and liposomal amphotericin‐B demonstrate significant reductions in amphotericin‐B‐induced nephrotoxicity without compromising their antifungal activities.^[^ ^45^ ^]^ Many novel drug delivery nanocarriers have since been introduced. These nanoparticles may be categorized into different classes based on their composition: phospholipid vesicles, non‐phospholipid vesicles, polymeric nanoparticles, polymeric micelles, solid lipid nanoparticles, nanostructured lipid carriers, nanoemulsions, and dendrimers. The most practical class of nanocarriers is the phospholipid vesicles, which include liposomes, deformable liposomes, ethosomes, transfersomes, and transethosomes.^[^ ^46^ ^]^

Denture stomatitis caused by *C. albicans* frequently occurs in patients wearing removable dentures. It is an erythematous inflammatory disease that is accompanied by burning sensation, unpleasant taste, and disturbance in masticatory function.^[^ ^47^, ^48^, ^49^ ^]^ Factors such as age, smoking, systemic diseases affect the severity of denture stomatitis.^[^ ^50^, ^51^ ^]^ Strategies to control denture stomatitis include oral hygienic education,^[^ ^52^ ^]^ phytotherapy,^[^ ^53^ ^]^ as well as controlling the predisposing factors.^[^ ^54^ ^]^ Denture adhesives are common materials used for enhancing the retention and stability or removal of oral prostheses. They are commercialized in the form of creams, strips, powder, and cushion formulations and can be included in mucoadhesive compositions.^[^ ^55^ ^]^ Mucoadhesion may be used to enhance antifungal activity by increasing the duration of drug release and bioavailability of antifungal formulations.^[^ ^56^, ^57^, ^58^ ^]^

Miconazole nitrate has been used extensively to treat superficial mucosal candidiasis.^[^ ^59^ ^]^ This antifungal agent is usually incorporated in oral gel formulations. When applied to the oral mucosa, these formulations have short contact times with the infected mucosa. This necessitates the use of high initial concentrations of the antifungal agent. In addition, these formulations do not have sustained release properties and frequent applications are necessary.^[^ ^60^ ^]^ Polymer‐based microscopical and nanoscopical delivery platforms have been designed to address the aforementioned deficiencies.^[^ ^61^ ^]^

**Figure 6:**
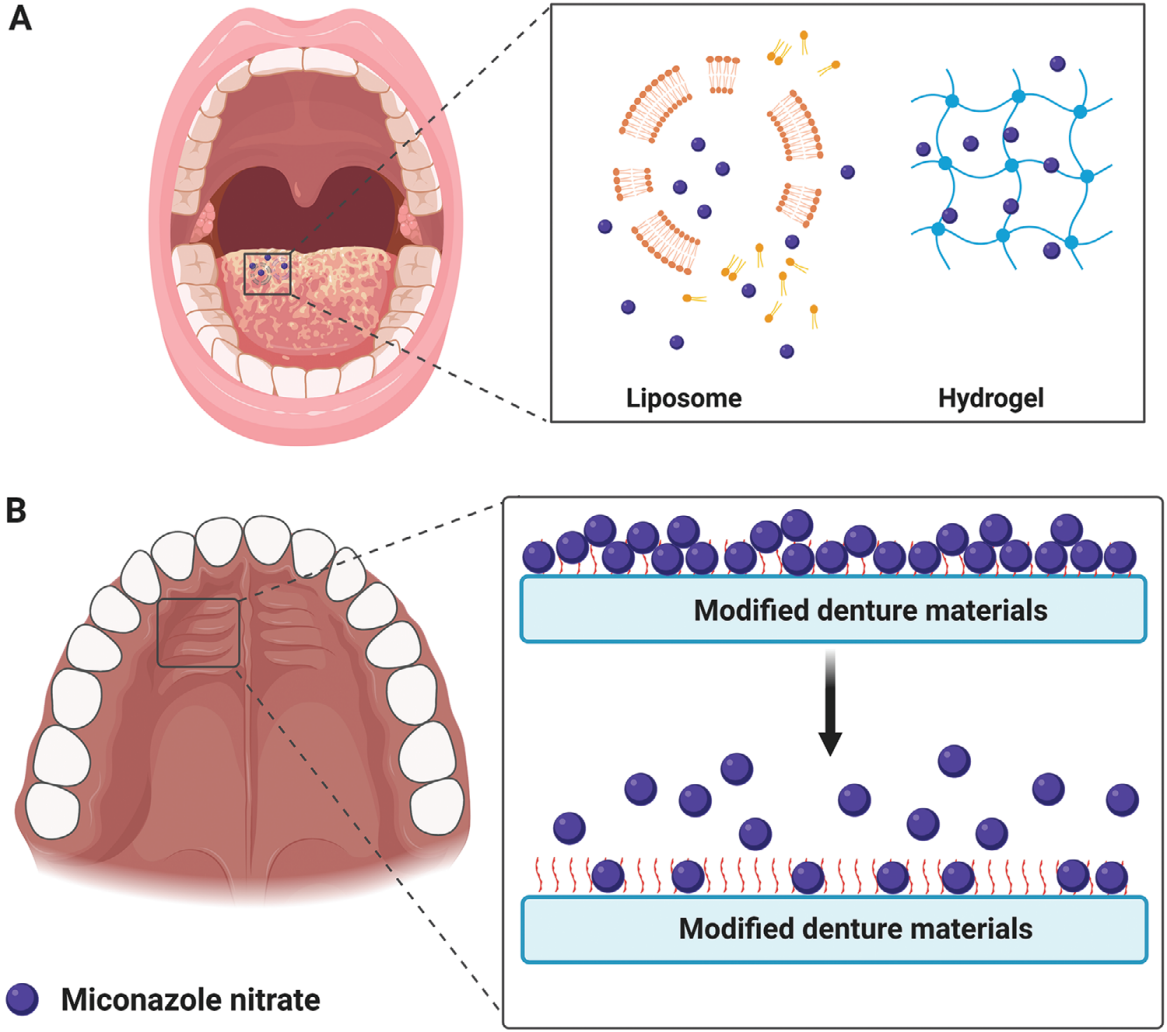
Antifungal delivery systems for treatment of oral candidiasis. A) Miconazole nitrate‐encapsulated liposome and hydrogel. B) Modified denture materials with prolonged miconazole nitrate release.

Different pH‐sensitive, mucoadhesive polymer‐based microparticles have been used to encapsulate miconazole nitrate. The pH‐sensitive microparticles release their cargo in a prolonged manner (**Figure** **6**). The composition is capable of improving the solubility of miconazole nitrate, with potent antifungal activities.^[^ ^59^, ^62^, ^63^ ^]^ Other antifungal drugs such as chlorhexidine (CHX) and nystatin have also been incorporated in resilient denture liners to inhibit the growth of *C. albicans*.^[^ ^64^, ^65^, ^66^ ^]^ These two antifungal agents demonstrated good biocompatibility and negligible toxicity in vivo, rendering promising candidates for treatment of denture stomatitis.^[^ ^67^ ^]^

Fluconazole is another antifungal agent that is used for site‐specific treatment of infections caused by *C. albicans*.^[^ ^68^, ^69^ ^]^ It is a synthetic antifungal agent derived from the group of triazole compounds.^[^ ^68^, ^70^ ^]^ Because of its side effects such as headache, nausea, liver disease, drug interaction, and the risk of drug resistance, it is necessary to utilize a suitable drug delivery system for its dispensary.^[^ ^69^ ^]^ Mucoadhesive nanoparticles have been synthesized to deliver fluconazole.^[^ ^71^ ^]^ Chitosan was used to coat the mucoadhesive nanoparticles to enhance their efficacy. Chitosan possesses many desirable properties, including its biocompatibility, non‐toxic nature, antifungal property, mucoadhesive property via attachment to negatively‐charged mucosal surfaces for extension of drug release duration.^[^ ^72^, ^73^ ^]^ In addition, the side effects of fluconazole were reduced considerably. Improved bioavailability of chitosan‐coated nanoparticles has been demonstrated in in vitro and in vivo experiments, with negligible manifestation of cytotoxicity.^[^ ^71^ ^]^

## 4 Antiviral Delivery

**Figure 7:**
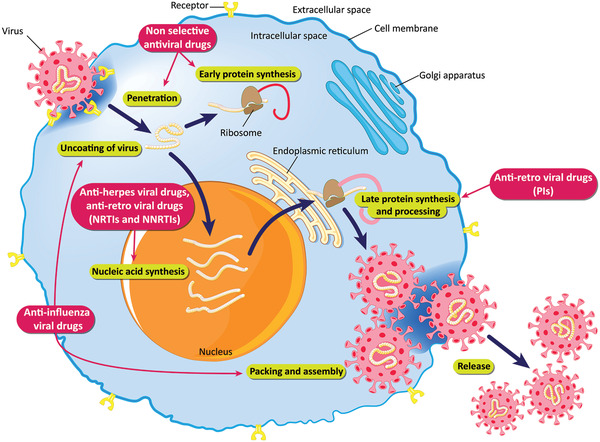
Schematic of the mechanisms of antiviral drugs. Reproduced with permission.^[^ ^78^ ^]^ Copyright 2018, ISFCP.

Antiviral drugs are active molecules used for treating viral infections.^[^ ^74^ ^]^ Although most of these agents are designed to target specific viruses, it is still possible to find drugs that are effective against a wide range of viruses.^[^ ^75^ ^]^ The mechanism of antiviral drugs is presented in **Figure** **7**.

Viral infections can involve the skin of the mouth as well as the oral mucosa, an example of which is represented by oral herpes infection. Oral herpes is caused by human herpes simplex virus 1 (HHV‐1) and causes pain on the lips, tongue, and the roof of the mouth.^[^ ^76^ ^]^ Other human herpes infections include the HHV‐2 virus that causes genital herpes, the HHV‐3 virus that causes chickenpox and herpes zoster, the HHV‐4 (Epstein‐Barr) and HSV‐5 (cytomegalovirus) viruses that cause infectious mononucleosis, the HHV‐6 and HHV‐7 viruses that cause roseola (a viral disease causing high fever and a skin rash in small children), and the HHV‐8 virus that causes Kaposi's sarcoma in people with acquired immunity deficiency syndrome.^[^ ^77^ ^]^ To date, systemic administration of antiviral drugs is still the most common strategy for treating oral virus infections.^[^ ^77^ ^]^ Very few local administration strategies are available in the literature and topical administration of antiviral agents, such as creams for labial herpes, is the only class of local drug delivery strategy available commercially.^[^ ^1^ ^]^

The use of nanoparticles for the delivery of antiviral drugs is an exciting field of research.^[^ ^75^ ^]^ Nanoparticles can tune the release kinetics of antiviral drugs, increase their bioavailability, control their dissolution rates, reduce their side effects, and lower the cost of treatment. Adoption of nanoparticle delivery platforms provides the possibility of targeting specific biological sites either passively or actively.^[^ ^74^ ^]^

## 5 Ion Delivery

Carbohydrate fermentation by *S. mutants* and *S. sobrinus* bacteria produce organic acids. The increased acidity triggers the release of calcium and phosphate ions from enamel and mineralized dentin. This demineralization process is counteracted by the activity of saliva, which contains bicarbonate ions for buffering the acidic changes and restoring the oral environment of normal pH value, as well as mineral ions that replenish the demineralized tooth surfaces with calcium and phosphate ions (remineralization). If this dynamic physiological balance is shifted such that the rate of demineralization is higher than that of remineralization, it will result in dental caries with consequential enamel dissolution.^[^ ^79^, ^80^, ^81^ ^]^ Ion delivery in the form of calcium, phosphate, and fluoride to suppress demineralization in the oral environment has been a major challenge for dental researchers for over a century. The following sections deal with delivery of different ions for dental applications.

### 5.1 Fluoride Delivery

Fluoride is a mineral‐source ion that prevents the growth of caries‐related bacteria and further acidification of the oral environment.^[^ ^80^, ^82^, ^83^ ^]^ It has been shown that hard tissue demineralization is reduced with increasing concentration of fluoride ions present in the saliva.^[^ ^84^ ^]^ By reacting with relatively more soluble hydroxyapatite, fluoride ions are incorporated in the hydroxyapatite lattice structure to produce more acid‐resistant fluorapatite. Fluoride ions also interfere with the metabolism of organic acid‐producing bacteria and prevent caries progression.^[^ ^80^, ^82^, ^83^ ^]^ A daily intake of 200 ppm of fluoride has been shown to prevent dental caries.^[^ ^85^ ^]^ To maintain the concentration of fluoride ions in the salvia, researchers have resorted to designing novel ion delivery systems that offer sustained ion release.

The use of microparticles and nanoparticles as delivering agents for fluoride ions have received much attention in recent years. The large surface‐to‐volume ratios of these particulates enable them to increase the amount of loaded ions. These particulates also possess the ability to release fluoride ions in a controlled manner that helps to maintain an optimized concentration of the ions and exert their protective effect over a longer period of time. In this context, nanoparticulate carriers are more effective than microparticulate carriers. In a recent study, NaF/chitosan microparticles were prepared by spray‐drying in the presence of glutaraldehyde as cross‐linker. The microparticles demonstrated continuous release of fluoride ions up to 360 min at pH 4 and pH 7.^[^ ^80^ ^]^ Fluoride ion‐containing ethylcellulose and gelatin microparticles with different sources of NaF, monofluorophosphate, and aminofluoride were prepared by hardening emulsion and spray‐drying methods; the in vitro results reported sustained release of the fluoride ions over a period of 8 h. Such a strategy presents a potential tool for the delivery of fluoride ions to dentin.^[^ ^86^, ^87^ ^]^

**Figure 8:**
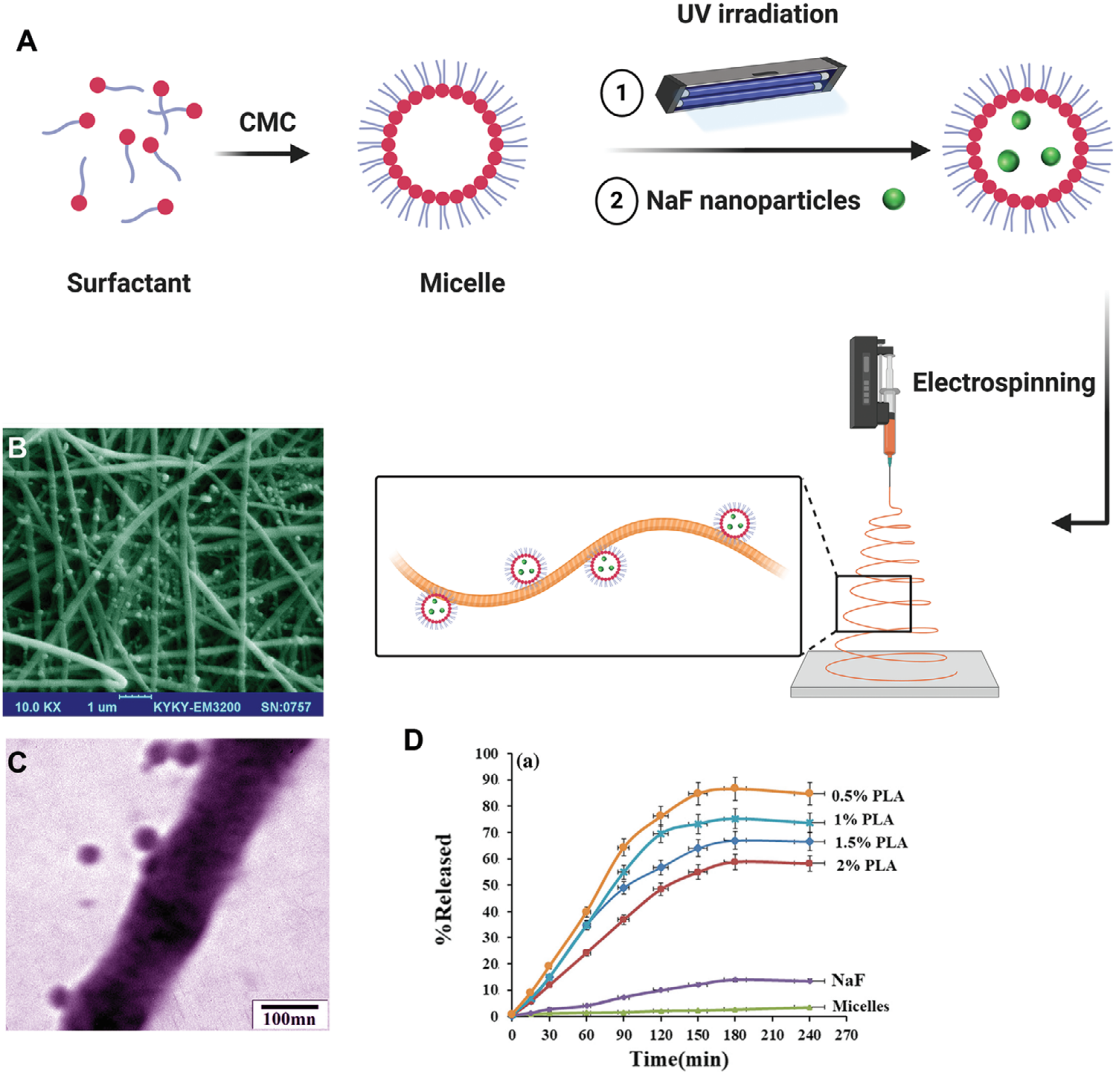
A) Synthesis of NaF nanoparticles followed by their loading into a polylactic acid scaffold via electrospinning. B) Scanning electron microscopy, and C) transmission electron microscopy images of the prepared NaF nanoparticles. D) Cumulative fluoride release from the polylactic acid scaffold compared to NaF in both free and micelles at 37 °C, pH: 7.4. CMC: critical micelle concentration, NaF: sodium fluoride, PLA: polylactic acid. (B–D) Reproduced with permission.^[^ ^91^ ^]^ Copyright 2020, Springer Nature.

The use of nanoparticles is a more efficient delivery approach than the use of larger‐sized particles because of the higher surface‐to‐volume ratio of nanoparticles. Calcium fluoride nanoparticles are 20 times more effective than traditional glass ionomer cement on remineralizing dentin.^[^ ^88^ ^]^ The anti‐cariogenic effect of CaF nanoparticles on *S. mutants* biofilms has been reported.^[^ ^89^ ^]^ In an in vitro study, chitosan/fluoride nanoparticles were prepared in the presence of sodium tripolyphosphate as a cross‐linking agent, with sustained release of fluoride ions from the nanoparticles. Fluoride release from the nanoparticles was increased in an acidic pH. The results suggest that these nanoparticles are capable of releasing fluoride ions in an acidic environment and expedite hard tissue remineralization.^[^ ^90^ ^]^ Calcium fluoride and lignocaine nanoparticles have been loaded in thiolated chitosan bioadhesive films for prolonged release of fluoride ions over 8 h.^[^ ^85^ ^]^ Recently, NaF nanoparticles have been prepared in the presence of surfactant and loaded on a polylactic acid nanoscaffold using electrospinning for delivery of fluoride ions to dentin (**Figure** **8**A). The nanoparticles ranged between 80 and 110 nm with the polylactic acid scaffold (Figure 8B,C). Sustained release of fluoride ions from the polylactic acid nanoscaffolds was observed, at a concentration of 5.0% mg mL^−1^, up to 4 h.^[^ ^91^ ^]^

Calcium and phosphate ions are depleted during bacterial acid‐induced hard tissue demineralization. Calcium phosphate nanoparticles doped with fluoride ions have been shown to form fluorapatite salts in water faster than undoped nanoparticles. In an in vitro dentin caries model, these nanoparticles can deliver calcium and fluoride ions to occlude dentinal tubules.^[^ ^92^ ^]^

One of the most practical applications of fluoride ions is their incorporation in mouthwashes. In people with a high risk of caries, these mouthwashes protect the teeth from acid demineralization via the production of fluorapatite.^[^ ^93^ ^]^ The use of fluoride‐containing mouthwashes enables demineralized tooth surfaces to be exposed intermittently to fluoride for long time periods to inhibit dental caries. In another study, mouthwashes containing different concentrations of chitosan nanoparticles were designed for sustained release of fluoride ions. Addition of 40 µg mL^−1^ chitosan to the mouthwash increased its viscosity and resulted in prolonged fluoride release to artificial salvia. The chitosan nanoparticles had no interaction with the rosins utilized in the mouthwash.^[^ ^94^ ^]^ *β*‐tricalcium phosphate nanoparticles functionalized with fluoride has been shown to be more effective than the traditional fluoride ion solution in remineralizin dentin.^[^ ^95^ ^]^

Bioactive glass (Bioglass) is a synthesized glass composition with controlled degradation that has demonstrated successful results in bone and tooth tissue engineering.^[^ ^96^ ^]^ In a pilot study, the levels of fluoride ions in the gingival crevicular fluid and saliva were significantly increased after 3 months of using fluoride‐containing bioglass in human volunteers.^[^ ^97^ ^]^ In a more thorough study, the bioavailability of fluoride ions from F‐containing bioglass was found to be equivalent to those present in high concentrations of sodium fluoride and amine fluoride.^[^ ^98^ ^]^

Layered double hydroxides (LDHs) are ionic layers with positively‐charged metal plates. The layered structure provides space for ion exchange and is used extensively in drug delivery systems.^[^ ^99^, ^100^, ^101^ ^]^ Positive metal plates are usually made up of M^+2^ and M^+3^ cations, which can also be loaded with negative ions such as fluoride ions. Fluoride‐incorporated LDH structure can be used as buccal mucoadhesive strips. An in vivo study conducted on 8 human volunteers showed that these structures are safe and efficient for prolonged release of fluoride ions to prevent dental caries via an ion‐exchange mechanism.^[^ ^84^ ^]^

Another application of fluoride is its differentiating effect on stem cells into bone and hard tissues. Previous studies have shown that the effect of fluoride ions on stem cells is dose‐dependent.^[^ ^102^, ^103^ ^]^ The toxicity of fluoride ions and their effects on differentiation of human dental follicle stem cells were investigated using nano silicate platelets in the presence or absence of fluoride ions. Results of bone regeneration indicated that nanosilicate platelets doped with fluoride enhanced osteogenic cell differentiation compared with platelets that did not contain fluoride ions.^[^ ^104^ ^]^ In another study, the effect of low‐level sodium fluoride on bone marrow mesenchymal stem cells was evaluated for the extent of wound healing and stem cell differentiation into osteoblasts after traumatic dental injury. The results indicated that 50 µm of sodium fluoride induced cell motility after 12 h stimulated osteoblast differentiation after 21 days.^[^ ^105^ ^]^

### 5.2 Ca and P Delivery

Calcium ions make up 99% of bone tissue. Administration of calcium carbonate, calcium lactate, or calcium gluconate helps to prevent osteoporosis and bone loss. The bulk of the minerals present in enamel is carbonated apatite, which comprises 10 calcium ions and 6 phosphate ions.^[^ ^106^, ^107^ ^]^ Hydroxyapatite, being biologically compatible, has been used in various formulations as a biomimetic agent against dental caries^[^ ^108^ ^]^ and dentin hypersensitivity.^[^ ^109^ ^]^

Calcium and phosphate‐based ion delivery systems such as hydroxyapatite,^[^ ^110^ ^]^ tricalcium phosphate,^[^ ^95^, ^111^ ^]^ and amorphous calcium phosphate (ACP) are promising agents for prevention of dental caries by increasing saturation of these ions in the oral environment.^[^ ^112^, ^113^, ^114^ ^]^ Polyamidoamine (PAMAM) dendrimers are a group of hydrophilic polymers with an ethylene‐diamine core and amidoamine branching structure that enable them to absorb calcium molecules.^[^ ^115^ ^]^ PAMAM dendrimers loaded with calcium and phosphate ions and have been used experimentally to prevent tooth decay. The loaded PAMAM dendrimer was effective for prolonged release of calcium and phosphate at low pH, with neutralization of the acidic environment and inhibition of dental caries.^[^ ^107^ ^]^

**Figure 9:**
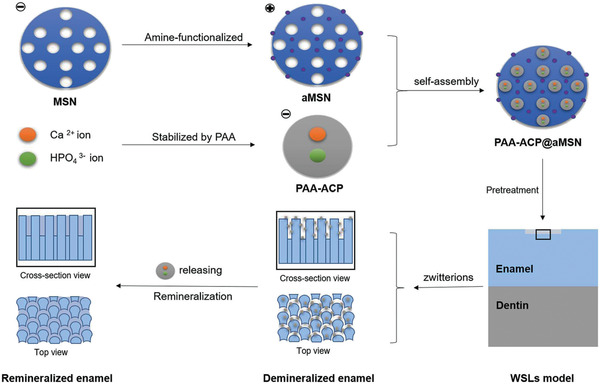
Synthesis of PAA‐ACP@MSN and its role in remineralization of demineralized enamel. PAA: polyacrylic acid, MSN: mesoporous silica, ACP: amorphous calcium phosphate, WSLs: white spot lesions. Reproduced with permission.^[^ ^121^ ^]^ Copyright 2020, Springer.

ACP nanoparticles do not have sufficient stability in the oral environment and are readily transformed into a crystalline form. This results in reduced bioavailability of calcium and phosphate ions for remineralization of tooth enamel. Polyacrylic acid has been used to increase the stability of ACP. Polyacrylic acid‐ACP was incorporated into mesoporous silica nanoparticles (MSNs) via electrostatic interaction. The system demonstrated sustained release of calcium and phosphate ions for remineralization of collagen fibrils in demineralized dentin.^[^ ^116^ ^]^ Casein phosphopeptide (CPP) is a cluster protein similar to salvia‐related stabilizing proteins. The phosphopeptide improves the bioavailability of calcium and phosphate ions by increasing the stability of ACP. CPP‐ACP has been shown to reduce tooth decay by releasing calcium and phosphate ions into the oral environment.^[^ ^117^ ^]^ CPP‐ACP has been used as an anti‐cariogenic electroneutral nanocomplex to promote remineralization in many commercial products such as toothpaste.^[^ ^118^, ^119^, ^120^ ^]^ Apart from CPP‐ACP, polyacrylic acid‐stabilized ACP incorporated into amine‐functionalized mesoporous silica (PAA‐ACP@aMSN) has also been shown to inhibit tooth decay by preservation of the microhardness and mineral content of the remineralized enamel. The structure of PAA‐ACP@aMSN is illustrated in **Figure** **9**.^[^ ^121^ ^]^

**Figure 10:**
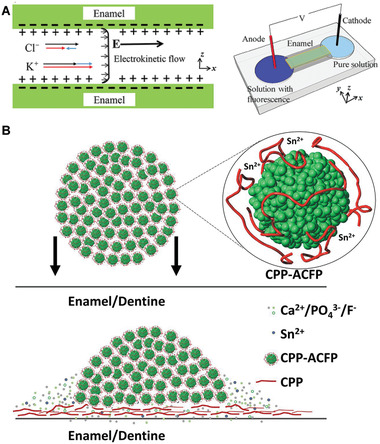
Recent studies on remineralization of tooth enamel. A) Nanofluidic transport by electrokinetic flow. Reproduced with permission.^[^ ^122^ ^]^ Copyright 2019, Springer, B) CPP‐ACP nanocomplex coupled with SnF_2_. CPP: casein phosphopeptide, ACFP: amorphous calcium fluorophosphate, SnF_2_: stannous fluoride. Reproduced with permission.^[^ ^123^ ^]^ Copyright 2019, Nature Publishing Group.

Enamel contains long narrow nano‐channels that facilitate ion infiltration. Nanofluidic transport of fluoride, potassium, calcium, and sodium ions into these enamel nano‐channels was induced by electrokinetic flow. Ion infiltration was confirmed using fluorescence and ion‐selective electrodes. Briefly, two enamel rod ends were exposed to two reservoirs, one filled with a solution containing a fluorescence probe, and the other with pure ionic solution (**Figure** **10**A). By applying an electric current, the ions are transferred from the left reservoir into the right reservoir by infiltrating into the enamel non‐channels.^[^ ^122^ ^]^ In another study, CPP‐ACP nanocomplexes were coupled with stannous fluoride to increase the stability of the nanocomplexes and ion release efficiency for the treatment of dental caries (Figure 10B).^[^ ^119^ ^]^

### 5.3 Sr Delivery

**Figure 11:**
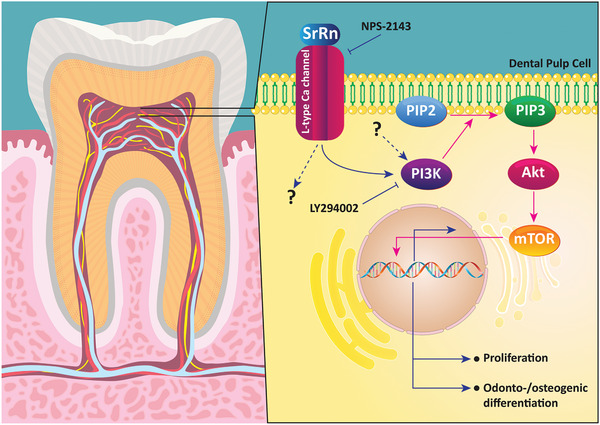
Release of Sr derived from SrRn into the cytoplasm of dental pulp cells stimulates osteogenic/odontogenic differentiation via the I3K/AKT signaling cascade. SrRn: Strontium ranelate, PIP2: phosphatidylinositol biphosphate, PI3K: phosphoinositide 3‐kinase, Akt: serine/threonine‐protein kinase B, mTOR: mammalian target of rapamycin.

Strontium (Sr^2+^) is a cation that stimulates the differentiation of mesenchymal stem cells to develop into bone tissue by suppressing the activity of osteoclasts as bone resorption cells.^[^ ^124^, ^125^ ^]^ In presence of Sr^2+^, mesenchymal stem cells have been shown to secrete bone‐related markers.^[^ ^126^, ^127^ ^]^ Strontium acetate has the potential to occlude dentin tubules and also improve dentin mechanical properties.^[^ ^128^ ^]^ Strontium ranelate (SrRn; the binding of two Sr^2+^ ions to ranelic acid) is a relatively novel anti‐osteoporotic drug^[^ ^129^ ^]^ that has been used for bone reconstruction.^[^ ^130^, ^131^ ^]^ The capacity of SrRn to replace calcium hydroxide and mineral trioxide aggregate as a pulp‐capping agent to induce mineralization in the dental pulp was investigated. The results indicate that SrRn enhances the proliferation of mouse dental papillae cells and human dental pulp stem cells, and promotes odontogenesis. The effect of SrRn on osteogenesis/odontogenesis is schematically illustrated in **Figure** **11**.^[^ ^132^ ^]^

**Figure 12:**
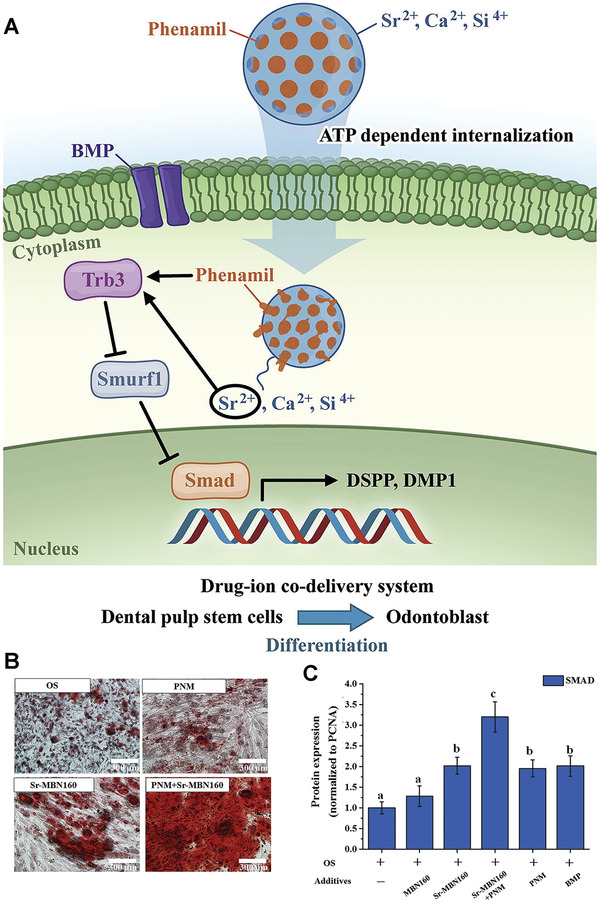
A) A drug‐ion codelivery system consisting of mesoporous bioglass nanoparticles loaded with phenamil and Sr^2+^ ions activates the BMP‐2/SMAD signaling pathway and stimulates differentiation of dental pulp stem cells into odontoblasts. B) Alizarin red S staining to show the formation of mineralized nodules in the cell culture plates. C) SMAD expression in the different experimental and control groups. BMP: bone morphogenetic protein; Trb3: tribbles homolog 3; Smurf1: smad ubiquitin regulatory factor 1*, *DSPP: dentin sialophosphoprotein, DMP1: dentin matrix protein 1, OS: osteoodontogenic, PNM: phenamil, Sr‐PNM: Sr‐doped phenamil, PCNA: proliferating cell nuclear antigen. Reproduced with permission.^[^ ^135^ ^]^ Copyright 2017, Elsevier.

Collagen/SrRn composite gels have been experimentally investigated as drug delivery systems for treatment of localized periodontitis.^[^ ^133^ ^]^ In an in vitro study, Sr^2+^‐doped mesoporous bioactive glass was used as a drug delivery system for dental tissue engineering. The presence of Sr^2+^ enhanced bone interconnectivity and sustained drug release.^[^ ^134^ ^]^ Other studies involving the use of Sr^2+^ delivery systems for dental tissue engineering are listed in **Table** **1**. The use of mesoporous bioglass nanoparticles as a hybrid drug/ion delivery system for hard bone tissue engineering has been reported. In that study, phenamil, a small molecule activator of bone morphogenetic protein (BMP), was loaded into Sr‐doped mesoporous bioglass nanoparticles for the stimulation of osteo/odontogenesis of human mesenchymal stem cells. Co‐delivery and concerted release of phenamil and Sr^2+^ resulted in activation of the BMP signaling pathway. The process of inducing differentiation of mesenchymal stem cells by the nanocarriers is shown in **Figure** **12**A. As shown in Figure 12B,C, bone matrix mineralization was increased when the stem cells were exposed to both Sr^2+^ and phenamil. Enhancement of SMAD protein expression resulted in promotion of the BMP‐2/SMAD signaling pathway.^[^ ^135^ ^]^ Table 1 shows some of the recent publications which have used nanocarriers containing ions for dental applications.

**Table 1:** Recent findings of the delivery of ions for dental application

Vehicle	Ions	Objective	Key finding	Ref
Collagen sponges	Sr^2+^	Produce spongious drug delivery systems for local periodontitis treatment	Enhanced effect of Sr on degradation time Increased drug release in the presence of Sr	^[^ ^133^ ^]^
Sr ion‐doped mesoporous bioactive glass	Sr^2+^	Synthesis of porous interconnected structures of strontium‐doped bioglass without using porogens	Sr enhances bone tissue interconnectivity The presence of Sr leads to sustained drug release SrBG is a promising drug delivery system for dental tissue engineering	^[^ ^136^ ^]^
Mesoporous phosphate‐based glass	Sr^2+^	Synthesis of an effective drug delivery system using different Sr concentration	Enhanced effect of Sr on degradation time Sustained release of loaded drug for bone regeneration	^[^ ^134^ ^]^
10% (w/w) strontium acetate solution	Sr^2+^	Dentin hypersensitivity treatment—investigation of duration (up to 28 days) and delivery method (brushed/soaked).	Increased tubule occlusion for normal root dentin	^[^ ^128^ ^]^
Sr‐doped *β*‐tricalcium phosphate	Sr^2+^	Preparation of strontium‐doped brushite and monetite cement	Improved drug release from cement Increased injectability in the presence of Sr	^[^ ^137^ ^]^
Chitosan‐amorphous calcium phosphate nanoparticles	Ca^2+^, PO_4_^3−^	Prevention and treatment of the enamel decalcification around orthodontic brackets	Smart pH‐responsive release of calcium ions Antibacterial and anti‐inflammatory effects	^[^ ^138^ ^]^
Cement containing amorphous calcium‐phosphate nanoparticles	Ca^2+^, PO_4_^3−^	Suppressing tooth decay	Possessing calcium and phosphate ion recharge/re‐release capability Caries inhibition	^[^ ^139^ ^]^
Sealer contains dimethylaminohexadecyl methacrylate, nanoparticles of silver, and nanoparticles of amorphous calcium phosphate	Ca^2+^, PO_4_^3−^	Kill bacteria inside root dentin and increase dentin hardness	Protect tooth root structures Prevent secondary endodontic infection	^[^ ^140^ ^]^
Combination of poly(amido amine) and calcium phosphate nanoparticle	Ca^2+^, PO_4_^3−^	Long‐term remineralization of dentin	Sustain ion re‐release and acid‐neutralization capability by nanoparticles Long‐term dentin remineralization Protect tooth structures	^[^ ^141^ ^]^
Nano‐size calcium‐phosphate lipid system	Ca^2+^, PO_4_^3−^	Dental regeneration	Down‐regulation of inflammation‐associated markers (IL‐1*β*, IL‐6, TNF‐*α*, COX‐2) of DPSCs Enhancment of the odontogenic differentiation potential of iDPSCs	^[^ ^142^ ^]^
Plate‐like *β*‐tricalcium phosphate nanoparticles	Ca^2+^, PO_4_^3−^	Determine the concentration‐dependent properties of nanoparticle on incipient enamel caries lesions	Time and concentration dependent mineralization	^[^ ^143^ ^]^
Sealant containing CaF_2_ nanoparticles and dimethylaminohexadecyl methacrylate	F¯	Release fluoride ions and antibacterial property	Strong antibacterial activity Caries inhibition Promoting enamel and dentin remineralization	^[^ ^144^ ^]^
Stannous fluoride‐functionalized *β*‐tricalcium phosphate nanoparticles	F¯	Evaluation of anti‐erosive effect	Nanoparticle improved anti‐erosive effect in comparison with fluoride solution Effective role in controlling the dentin erosion	^[^ ^145^ ^]^
Fluorinated bioactive glass nanoparticles	F¯	Prevention of white spot lesions	Antibacterial and anti‐demineralization effects Potential application in orthodontic resins	^[^ ^146^ ^]^
Nano‐CaF_2_ orthodontic cement	F¯	Prevent formation of white spot lesions in enamel Provide long‐term and high levels of F release	Sustained fluoride ion release and recharge Inhibition of the formation of white spot lesions around orthodontic brackets.	^[^ ^147^ ^]^
Calcium fluoride nanocomposite	Ca^2+^, F¯	Prevent recurrent caries	Strong antibacterial and ion release capabilities Reduce biofilm production Inhibit recurrent caries Increase restoration longevity	^[^ ^148^ ^]^

Abbreviations. DPSC: dental pulp stem cells, IL‐1*β*: interleukin‐1 beta, IL‐6: interleukin‐6, TNF‐*α*: tumor necrosis factor‐alpha, COX‐2: cyclooxygenase‐2, iDPSCs: inflamed dental pulp stem cells, CaF_2_: calcium fluoride.

## 6 Gene and Protein Delivery

### 6.1 Peptide Delivery

One of the most common drugs for controlling tooth decay is CHX. Although CHX is an excellent antibacterial agent, its application is associated with side effects such as mouth irritation, tooth staining, and dry mouth.^[^ ^149^ ^]^ As an alternative, antimicrobial peptides (AMPs) demonstrate potential in the treatment of biofilms that cause tooth decay.^[^ ^150^ ^]^ AMPs cause microbial destruction by applying electrostatic changes to the membrane of microbial cells followed by creating cavities and increasing osmotic pressure.^[^ ^149^, ^151^ ^]^ Despite the excellent properties of AMPs compared with antibiotics, their high cost of production, complex downstream processes, and instability in saliva are challenges that hinder their commercialization.^[^ ^149^, ^152^ ^]^ A recent approach to overcoming the high cost of manufacturing AMPs is to utilize natural resources known as plant‐made AMPs (PMAMPs). Linear AMPs usually have lower antimicrobial activities and stability than AMPs with complex secondary structures. Retrocyclin and protegrin‐1 with cyclic/hairpin structures are more stable and efficient in comparison to the linear forms.^[^ ^149^ ^]^ In a study, acyclic PMAMPs were developed as an inexpensive method for combating dental diseases caused by microbial biofilm. These PMAMPs destroy microbial biofilms by perforating bacterial membranes, with antimicrobial activities comparable to CHX. The cationic property of protegrin‐1 also permits electrostatic interaction with the hydroxyapatite present on tooth surfaces and enhances stability.^[^ ^149^ ^]^ An in vivo study reported that GH12, a cationic amphipathic *α*‐helical AMP, reduced the production of exopolysaccharides by *S. mutans* biofilms and decreased the acidity of the microenvironment.^[^ ^153^ ^]^ Peptide polyphemusin I is another natural AMP isolated from horseshoe crabs that contain 17 to 18 amino acids.^[^ ^154^ ^]^ In vitro and in vivo results confirmed the antimicrobial properties of the peptide polyphemusin I. This AMP may be used in toothpaste and commercial mouthwashes.^[^ ^155^ ^]^ Natural AMPs gradually become alternatives to antibiotics for the treatment of infectious diseases.

### 6.2 Delivery of Growth Factors

Growth factors are a group of bioactive agents with the ability to attach to specific transmembrane receptors on the surface of cells. Integration of growth factors to their receptors results in the activation of different intracellular pathways that account for cell differentiation, proliferation, and migration that are essential for tissue regeneration.^[^ ^156^, ^157^ ^]^

Tricalcium silicate‐based bioceramics, especially mineral trioxide aggregate, have been used to stimulate reparative dentin deposition in exposed dental pulps. The release of siliceous ions at the exposure site stimulates mesenchymal stem cells in the dental pulp to differentiate into odontoblast‐like cells. The latter secrete bone‐like material to wall off the pulpal exposure from the pulpal side. In tissue engineering, signals in the form of growth factors may be delivered to increase repair/regeneration efficacy by an engineered biological scaffold.^[^ ^158^ ^]^ Dental tissue engineering is one of the new approaches employed in regeneration of necrotic dental pulps in teeth with immature root formation. The regenerative material consists of three components: stem cells, scaffold, and bioactive cues in the form of growth factors or cytokines. Human dental pulp stem cells are a specific group of mesenchymal stem cells harvested from the tooth pulp. They are multipotent and have the capacity to differentiate into bone, teeth, blood vessels, adipose tissues, and neurons.^[^ ^159^ ^]^ Regular release of growth factors is essential for the natural healing of tissue after injury. Direct injection of growth factors into a defect site has resulted in effective healing. However, the high cost associated with the use of growth factors, structural limitations, and the adverse effects of high doses have limited the direct use of these molecules for clinical studies.^[^ ^160^ ^]^ To promote dental differentiation, growth factors delivered by specific vehicles may be used to control the specific signaling pathway and gene expression of stem cells within a site of injury and stimulate them to differentiate into bone or dentin forming cells.^[^ ^161^ ^]^

BMPs are a group of growth factors that are of particular importance in bone tissue engineering.^[^ ^162^, ^163^ ^]^ The effects of BMP‐4^[^ ^164^ ^]^ and BMP‐9^[^ ^165^ ^]^ on stem cell differentiation has been well reported in dental tissue engineering. BMP‐2 has been shown to induce bone/tooth differentiation.^[^ ^159^ ^]^ The use of BMP‐2 in dental implants has been reported to stimulate the migration of dental follicle stem cells.^[^ ^166^ ^]^ Despite their highly‐inductive properties, growth factors are unstable at 37 °C with short shelf lives. Hence, it is necessary to utilize smart delivery systems to control their release and increase their effectiveness. BMP‐2 had been loaded into calcium silicate scaffold for sustained release to stimulation the induction of mesenchymal stem cells.^[^ ^158^ ^]^ In another study, BMP‐2 was loaded into a titanium structure with poly(lactic*‐co‐*glycolic) acid layers and used in dental implants for controlled release of the growth factor.^[^ ^167^ ^]^ A printable 3D hydrogel was prepared with a bio‐ink consisting of gelatin/methacrylate and a synthetic BMP‐2‐mimicking molecule. A short amino acid sequence derived from natural BMP‐2 was synthesized in vitro. The purpose of using this molecule was to accelerate the differentiation of encapsulated MSCs within the printed structure into tooth‐forming cells. Such a system holds promise for dental tissue engineering.^[^ ^168^ ^]^ Stromal cell‐derived factor 1 *α* (SDF‐1*α*) is a chemokine that causes mesenchymal stem cells to migrate from specific stem cell niches to the site of injury.^[^ ^169^, ^170^ ^]^ An in vivo study demonstrated that SDF‐1*α* promoted differentiation of dental pulp stem cells, which was followed by pulp regeneration.^[^ ^171^ ^]^ The synergistic effect of BMP‐2 and SDF‐1*α* on human stem cells from the apical papilla was demonstrated by incorporating the growth factor and the cytokine into an injectable hydrogel and implanted into a necrotic tooth. Hard tissue regeneration was confirmed in vivo.^[^ ^172^ ^]^

Fibroblast growth factor (FGF) plays a hemostatic role in post‐injury tissue repair and has been extensively studied in dental tissue engineering.^[^ ^173^, ^174^ ^]^ FGF8, a member of the paracrine‐acting FGF family, has been shown to orchestrate the migration, proliferation, and differentiation of dental pulp stem cells.^[^ ^175^ ^]^ FGF8 has the potential to induce the earliest dental markers such as Lhx6 and Lhx7.^[^ ^176^ ^]^ Recently, it was reported that early delivery of exogenous FGF2 to dental pulp resulted in accelerated odontoblast differentiation.^[^ ^177^ ^]^

Integrated regeneration of tooth tissue requires the creation of an angiogenic network for the transfer of nutrients and oxygen between cells and the removal of by‐products. It has been shown that amplification of the Wnt signaling pathway leads to the vascularization of human dental pulp stem cells.^[^ ^178^ ^]^ Vascular endothelial growth factor (VEGF) is another growth factor in which its differentiation and angiogenic properties have been extensively studied on dental pulp stem cells.^[^ ^179^, ^180^ ^]^ In an in vitro study, VEGF was loaded into a temperature‐sensitive chitosan/glycerol phosphate hydrogel and its effect on dental pulp stem cells was compared with the use of VEGF alone. The VEGF‐loaded hydrogel was capable of controlled release of the growth factor to stimulate angiogenesis.^[^ ^181^ ^]^ The positive effect of the synergistic use of BMP‐2 and VEGF on the simultaneous effect of differentiation of dental pulp stem cells and angiogenesis has been reported.^[^ ^182^ ^]^ Controlled release of growth factor‐encoding plasmids (nanoplexes) instead of the use of free growth factors represent more efficient approaches. Nanoplexes consisting of electrostatic interactions between phosphate in the plasmids and polyethyleneimine groups have been extensively studied in gene delivery systems.^[^ ^183^, ^184^ ^]^ Polyethyleneimine nanoplexes and plasmids encoding BMP and FGF had been incorporated into a collagen scaffold for use as dental cement. Ex vivo results indicate that the aforementioned system can compete with mineral trioxide aggregate in stimulating the differentiation of human dental pulp stem cells in endodontics.^[^ ^185^ ^]^ All these reports confirm the importance of using growth factor delivery system for tooth regeneration.

Beyond pharmacological activity and studies on possible toxic products derived from nanomaterials key importance should also be given to technological issues and in particular manufacturing method, that up to now represents one of the main challenges in nanoparticles translation to clinic.^[^ ^186^ ^]^ Indeed scale‐up from a few grams produced in the laboratory to several kilos on an industrial setup is required. Therefore reproducible, easily scalable processes following the good manufacturing practice principles are important prerequisites.^[^ ^187^ ^]^ Strongly connected to this is the thorough characterization of the final product. In the case of polymer NPs, the therapeutic outcome is indeed related to the complex interactions of composition and microstructure. Therefore, it is essential to gain information about the physical characterization, the size distribution through dynamic light scattering, the surface zeta‐potential, and the NP shape or morphology via microscopy techniques. From this detailed analysis, few key parameters should be considered as reporters for formulation efficacy, in order to develop a robust quality control procedure necessary for NPs translation to clinics.^[^ ^188^ ^]^

## 7 Applications

**Figure 13:**
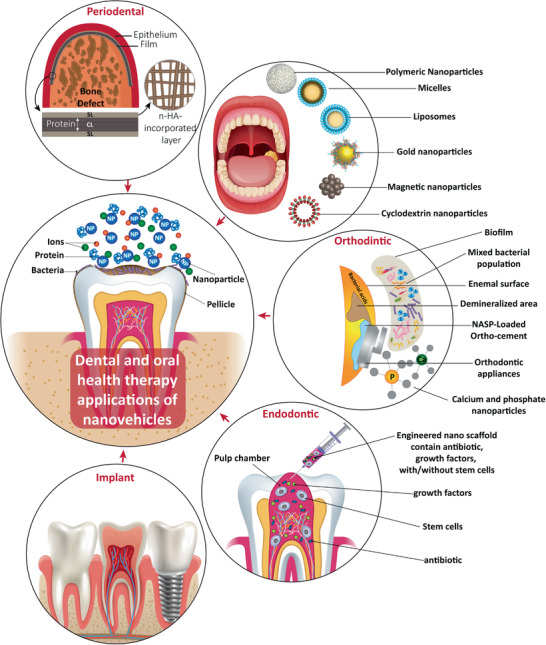
Oral applications of nanomaterials. HA: hydroxyapatite, NACP: nano‐sized amorphous calcium phosphate.

Nanomaterials may be used in different dental specialties (**Figure** **13**). Some of these applications will be covered in the following sections.

### 7.1 Endodontics

The objective of root canal treatment is to eliminate microorganisms and their by‐products from the infected root canal system. Achieving this goal involves cleaning and shaping of the canal space to eradicate bacterial and fungal biofilms, disinfecting the root canal system with antimicrobial and hard and soft tissue dissolving irrigants, and the placement of intracanal medicaments and subsequently permanent plastic fillings to inhibit bacterial re‐growth and to prevent microleakage.^[^ ^189^ ^]^ Nanoparticles have been introduced for advanced disinfection of the root canal system owing to their broad‐spectrum antibacterial and anti‐biofilm activities.^[^ ^190^ ^]^ One of the frequently investigated nanoparticles in endodontics is chitosan nanoparticles because of their intrinsic antimicrobial properties and their capability to encapsulate drugs/bioactive molecules.^[^ ^191^ ^]^ Adherence of *Enterococcus faecalis* to canal walls was significantly reduced when root canal dentin was treated with chitosan nanoparticles for root canal disinfection.^[^ ^192^ ^]^

Chitosan has also been used as a coating for poly(lactic*‐co‐*glycolic) acid for the delivery of ciprofloxacin for treating root canal infections. The effect of ciprofloxacin loaded with the polymeric nanocarriers was evaluated against *E. faecalis*, as a type of bacteria that is usually found in teeth with apical periodontitis that do not heal after root canal treatment. The antibacterial and anti‐biofilm capability of this nanosystem renders it potentially useful for use as an interappointment intracal medicament.^[^ ^193^ ^]^

Nanoparticle‐based photosensitizers have been found to augment the antimicrobial effect of photodynamic therapy. In an in vitro study, methylene blue dye‐loaded poly(lactic*‐co‐*glycolic) acid nanoparticles were used in combination with photodynamic therapy to eradicate *E. faecalis* biofilms, with improved bacterial phototoxicity.^[^ ^194^ ^]^ Similarly, the use of chitosan functionalized with rose bengal stain resulted in significant elimination of biofilms with reduced cytotoxicity to fibroblasts.^[^ ^195^ ^]^

Obturation of the root canal space with gutta‐percha and a sealer is intended for sealing of the debrided canal and prevent bacterial infection/re‐infection of the periapical tissue.^[^ ^196^ ^]^ Attempts have been made to improve the antibacterial properties of root canal sealers by incorporating different nanoparticles, but without adversely affecting the mechanical properties of the sealer. An interesting approach was proposed by synthesizing a sealer that contained propolis incorporated in polymeric nanoparticles. The polymeric nanoparticles provided sustained propolis delivery without fluctuations in its rate of release. The experimental sealer demonstrated cytotoxic properties that are comparable to a commercially available sealer, and possesses antimicrobial activities against *E. faecalis*, *S. mutans*, and *C. albicans*.^[^ ^197^ ^]^ Likewise, silver nanoparticles were incorporated into dimethylaminohexadecyl methacrylate to create a root canal sealer with good sealing, flow, and anti‐biofilm properties.^[^ ^198^ ^]^ Good antibacterial properties have also been obtained by directly adding 2.5% dimethylaminohexadecyl methacrylate and 0.15% silver nanoparticles into a commercially available epoxy resin‐based root canal sealer.^[^ ^199^ ^]^

Root canal treatment is challenging because of the complex anatomy of the root canal system and the difficulty involved in complete destruction of biofilms from the canal walls. Accordingly, advanced techniques involving nanotechnology have been developed to overcome the shortcomings of conventional procedures. The application of nanoparticles in the field of endodontics has potential to eradicate persistent endodontic pathogens.

### 7.2 Restorative Dentistry

Dental caries is by far the most frequent reason for tooth loss and is caused by bacterial biofilm. After teeth brushing, the early colonizers that can be found on tooth surface are non‐pathogenic bacteria such as *S. sanguinis* and *S. gordonii*. However, poor oral hygiene and inadequate diet can cause dysbiosis and lead to the formation of pathogenic biofilm, within which *S. mutans* plays an important role in production of glucan matrix, making the biofilm a solid formation difficult to remove. The production of acid within biofilms causes disruption of enamel integrity and, within time, leads to the formation of caries lesion.^[^ ^200^ ^]^ Treatment of dental caries involves removal of the infected carious tissue. This is followed by replacing the lost tissue with a metal, resin composite, or ceramic restoration that is bonded to the remaining tooth structure using a dental adhesive system.^[^ ^201^ ^]^ Currently, two different strategies can be used on dentin tissue during resin bonding procedures: etch‐and‐rinse and self‐etch technique. Regardless of the approach used, the application of adhesive system results in the formation of hybrid layer (HL) – a structure that is composed of demineralized collagen fibrils reinforced by resin matrix. The key to successful and long‐term bonding lies in the stability and integrity of collagen fibrils within the HL. However, the application of adhesives results in incomplete hybridization of the dentin substrate, leaving behind unprotected collagen fibrils surrounded by water, that are prone to hydrolytic degradation by endogenous enzymes. Consequently, as a result of degradation of the HL's components, micro‐cracks and secondary caries can occur after tooth restoration.^[^ ^202^ ^]^ Hence, it is imperative to develop dental materials with antibacterial properties that show good clinical results.^[^ ^203^, ^204^ ^]^ For this purpose, different nanoparticles with antibacterial and self‐healing properties have been incorporated into dental adhesive systems. One of the approaches to reduce biofilm formation on restorations is to apply commercially available dentin adhesives that contain 10‐methacryloyloxydodecylpyridinium bromide.^[^ ^205^ ^]^ Newly developed dental adhesives containing microcapsules, dimethylaminohexadecyl methacrylate, and ACP nanoparticles demonstrated optimal results in terms of phosphate ion recharge, protein‐repellent, and antibacterial properties.^[^ ^206^, ^207^ ^]^

Similar results have been achieved by combining ACP nanoparticles with 2‐methacryloxylethyl dodecyl methyl ammonium bromide,^[^ ^208^ ^]^ as well as combining ACP nanoparticles with 2‐methacryloyloxyethyl phosphorylcholine^[^ ^209^ ^]^ in dentin adhesive systems. Experimental adhesive systems containing 50–80% (v/v) of nitrogen‐doped titanium dioxide nanoparticles displayed satisfactory antibacterial properties against *S. mutans* biofilms which are responsible for secondary caries.^[^ ^210^ ^]^ Attempts have also been made to incorporate silver nanoparticles into commercially available dentin adhesive systems. Addition of Ag NPs in concentrations of 250 ppm into an adhesive produced superior antibacterial results, with dentin bond strength that are at par with commercial adhesive even after 6 months of water storage.^[^ ^211^ ^]^

Apart from adding nanoparticles to adhesive systems, recent studies have also investigated the possibility of integrating nanoparticles into restorative materials. ACP nanoparticles with and without addition of dimethylaminohexadecyl methacrylate have been incorporated into resin composite materials. Their anti‐bacterial effect, potential of remineralization, and mechanical properties were evaluated. The resin composite possessed mechanical properties that were similar to commercially available composites. With respect to remineralization potential, high levels of Ca and P were released over time. Incorporation of dimethylaminohexadecyl methacrylate into the ACP nanoparticle‐containing composite did not impair its mechanical or remineralization properties; its incorporation significantly improved the anti‐bacterial potential by reducing the number of bacteria and production of lactic acid.^[^ ^212^, ^213^ ^]^

CHX, an antimicrobial agent used extensively in dentistry, can be successfully blended within adhesive systems.^[^ ^214^ ^]^ The development of an adhesive with CHX‐containing nanoparticles is an interesting strategy for combating secondary caries in the future.

A nanocomposite indicated for restoring class V lesions (located in the root part of the tooth and in close contact with periodontal tissues) was synthesized with the addition of not only ACP nanoparticles and dimethylaminohexadecyl methacrylate, but also silver nanopaticles and 2‐methacryloyloxyethyl phosphorylcholine. The goal of adding silver nanoparticles was to enhance antimicrobial properties. 2‐methacryloyloxyethyl phosphorylcholine with protein‐reliant characteristics was incorporated with the intention of reducing bacterial adhesion to the implant surface. The new composite possessed potential antibacterial effect against periodontitis‐related pathogens, with satisfactory bond strength values.^[^ ^215^ ^]^ Silver nanoparticles embedded within lactose‐modified chitosan demonstrated reduced the formation of mature *S. mutans* biofilms and such an ability was found to be dependent on the concentration of silver nanoparticles within the coating layer.^[^ ^216^ ^]^

### 7.3 Oral Surgery and Implantology

Dental implants are commonly used in dentistry to replace missing teeth, with a 90–95% success rate. Failure of dental implants occurs because of inaccurate planning, improper surgery, and prosthesis application, material failure, and lack of maintenance. Infection is the most serious complication among the reasons for dental implant failure. The incidence of peri‐implant mucositis and peri‐implantitis infections characterized by bacterial accumulation on the implant surface is increasing dramatically. Hence, different strategies have been developed to enhance the antibacterial effect of implants. One promising strategy for providing strong fixation and low failure of dental implants is surface coating with nanoparticles to disrupt bacterial colonization, so that osseointegration may be successfully induced in the absence of bacterial infection.^[^ ^191^, ^217^, ^218^ ^]^

Bone mineral density and bone formation were enhanced by embedding silver nanoparticles on the implant surface, without causing damage to tissues surrounding the dental implants. Furthermore, no toxicity to the viability and differentiation of bone marrow mesenchymal stem cells was observed with this coating method.^[^ ^219^ ^]^ Another study reported that implant coatings consisting of silver nanoparticle‐filled hydrogen titanate (H_2_Ti_3_O_7_) nanotubes demonstrated more potent antibacterial activity, stronger osteogenetic potential, and low toxicity for stem cells.^[^ ^220^ ^]^ Similarly, silver nanoparticle‐doped Ti_6_Al_4_V alloy surfaces exhibited significant antibacterial activity against *Porphyromonas gingivalis* and *Prevotella intermedia*, with excellent biocompatibility.^[^ ^221^ ^]^

### 7.4 Prosthodontics and Orthodontics

Prosthodontics is crucial for replacement of lost teeth, restoration of oral function, and facial appearance. Treatment options include removable partial or complete dentures, fixed tooth‐supported or implant‐supported prostheses. Nanoparticles have gained attention in prosthodontics and have been incorporated into ceramics, resins, and metals.^[^ ^222^ ^]^ Polymethyl methacrylate (PMMA) is commonly used as denture base material because of its esthetic properties, biocompatibility, lightweight, low‐cost, and stability. On the other hand, PMMA‐based dentures are easily colonized by bacterial and fungal biofilms and suffer from frequent fractures.^[^ ^223^ ^]^ Several PMMA modification strategies have been developed to overcome these drawbacks.

Nanoparticles have been used to enhance the surface hydrophobicity of PMMA‐based dentures to provide antimicrobial activity. Incorporation of silver nanoparticles into a denture base acrylic resin was reported and it had no effect on the adherence of *C. albicans* and biofilm formation.^[^ ^224^ ^]^ However, it has been shown that silver nanoparticles had antifungal activity and reduced *C. albicans* formation.^[^ ^225^, ^226^ ^]^ Incorporation of poly(diallyldimethylammonium) chloride nanoparticles into PMMA produced antibacterial effect against *E. coli, Staphylococcus aureus*, and *C. albicans*.^[^ ^227^ ^]^ Similarly, PMMA denture acrylic containing platinum nanoparticles had a significant bacterial anti‐adherent effect.^[^ ^228^ ^]^

Improving the antibacterial capacity of fixed orthodontics appliances has been crucial in dentistry to prevent development of white spot lesions, which is the most common side effect associated with placement of orthodontic brackets. An orthodontic adhesive incorporating curcumin‐doped poly(lactic*‐co‐*glycolic acid) nanoparticles was evaluated for its anti‐biofilm efficacy against *S. mutans* biofilms. The effectiveness of the drug‐loaded nanocarriers was confirmed and may be used as an antibacterial and anti‐biofilm orthodontic adhesive.^[^ ^229^ ^]^

### 7.5 Periodontics

Periodontal disease is a common inflammatory disorder induced predominantly by Gram‐negative anaerobic bacteria such as *P. gingivalis*, *Aggregatibacter actinomycetemcomitans*, *Tannerella forsythia*, and *Treponema denticola*. If left untreated, periodontal diseases destroy the tissues surrounding and supporting the tooth, eventually causing the loss of tissue, bone, and finally the tooth. Treatment of periodontal diseases is crucial for the maintenance of oral and general health of the patient.^[^ ^230^ ^]^

The periodontal pocket provides a natural reservoir that is easily accessible to accommodate a dispensing device. Gingival crevicular fluid is an exudate discharged by the periodontal pocket that provides a medium for drug release and for its spreading to adjacent tissues. Intra‐pocket drug delivery systems have gained attention because of their superior features such as maintaining effective concentrations of antibiotics in the gingival crevicular fluid for an extended time and causing more benign side effects. Several polymers have been investigated as drug carriers because of their biocompatibility, stability, non‐toxicity, and biodegradability.^[^ ^231^ ^]^ Triclosan‐loaded nanoparticles prepared from poly(D,L‐lactide*‐co‐*glycolide), poly(D,L‐lactide), and cellulose acetate phthalate have been used to develop a novel delivery system for the treatment of periodontal disease. These triclosan‐loaded nanoparticles behaved as a homogeneous polymer matrix‐type delivery system in reducing gingival inflammation.^[^ ^232^ ^]^

Another method of intra‐pocket delivery is films that are formed by solvent‐casting or direct milling. Solvent‐cast poly(lactic*‐co‐*glycolic acid) films were used in a layer‐by‐layer technique to develop a localized and controlled drug delivery system. This system exhibited antibacterial activity against *P. gingivalis*, with exhibiting in vitro biocompatibility.^[^ ^233^ ^]^ Polymeric PolymP‐n Active nanoparticles combined with silver and doxycycline exhibited enhanced antimicrobial activity against *Streptococcus oralis*, *Actinomyces naeslundii*, *Veillonella parvula*, *Fusobacterium nucleatum*, *P. gingivalis* and *Aggregatibacter actinomycetemcomitans*.^[^ ^234^ ^]^

Regenerative procedures such as guided tissue regeneration (GTR) have taken advantage of the novel technologies based on tissue engineering. Bone grafting materials derived from biological tissues or from synthetic origins are used to augment the alveolar bone and maintain space for future implants. Biocompatible materials have been investigated due to the drawbacks of osseous grafts, such as the need for a second surgery, limited availability (autografts), disease transmission, high resorption risks (allografts), antigenicity, and unpredictable results (xenografts).^[^ ^235^ ^]^ Biocompatible materials for grafting should have superior features such as being osteoconductive, sustaining the load applied on the defect site as new bone grows, and self‐dissolving without any toxic effects.

Polytetrafluoroethylene (PTFE) membranes for GTR are commonly used because of its porous microstructure that allows connective tissue ingrowth. Some studies reported that ePTFE membranes provide superior regeneration of periodontal tissues after healing.^[^ ^236^ ^]^ Antibiotics and metal/metallic oxide such as silver, zinc, copper, and zinc oxide nanoparticles have been incorporated into the GTR membranes to improve periodontal healing. Incorporation of metronidazole into polycaprolactone nanofiber membranes produced clear inhibition zones around the GTR membranes.^[^ ^237^ ^]^ In a study, electrospun composite fibers prepared from mixing poly(DL‐lactide*‐co‐*e‐caprolactone) and poly(D,L‐lactide) with gelatin were loaded with hydroxyapatite nanoparticles to enhance osteoconductive activity. Metronidazole was used to eliminate periodontal pathogens. This novel functionally‐raded membrane possessed better potential to overcome the disadvantages of currently available membranes.^[^ ^238^ ^]^

Poly(*ε*‐caprolactone)–poly(ethylene glycol)–poly(*ε*‐caprolactone) (PCL–PEG–PCL) is linear triblock copolymer used for guided bone regeneration because of its biocompatibility and biodegradability. Nanohydroxyapatite had been incorporated into electrospun PCL‐PEG‐PCL membranes. The tensile strength decreased with increasing mineral content but there was no adverse effect on the viability of osteoblasts. Developing of a 3‐layer scaffold that a chitosan/poly(lactic*‐co‐*glycolic acid)/nano‐sized bioactive glass layer loaded with cementum protein 1, a chitosan/poly(lactic*‐co‐*glycolic acid) layer loaded with FGF 2, and a chitosan/poly(lactic*‐co‐*glycolic acid)/nano‐sized bioactive glass layer loaded with platelet‐rich plasma expedited periodontal healing and new alveolar bone deposition.^[^ ^239^ ^]^ A novel membrane consisting of CaP nanoparticles incorporated in a silk fibroin‐PCL‐PEG‐PCL electrospun layer and a PCL membrane layer has been developed for guided bone regeneration. The membrane demonstrated better cell adhesion and proliferation of dental pulp stem cells, with remarkable improvement in tensile strength.^[^ ^240^ ^]^

Silk fibroin is another polymer frequently employed for guided bone regeneration. It has superior features such as biocompatibility, biodegradability, as well as oxygen and water vapor permeability. Silver fibroin membranes were useful for guided bone regeneration of several types of bone defects.^[^ ^241^ ^]^ Nanofibrous silver fibroin membranes developed by electrospinning demonstrated better new bone regeneration, reduced inflammatory reaction, and improved mechanical stability compared with unfilled conventional collagen membranes.^[^ ^242^ ^]^

### 7.6 Regenerative Dental Medicine

Several branches of dentistry have already combined regenerative medicine with existing dental practices such as socket preservation, scaffolds, dental implants, and PRGF (protein‐rich growth factor) techniques. Scaffolds have a crucial role in regenerative dental medicine. Many bone graft materials and substitutes are composed of scaffolds. Studies have focused on finding the ideal scaffold type to facilitate growth, cell spreading, adhesion, and differentiation of mesenchymal stem cells.^[^ ^243^ ^]^

**Figure 14:**
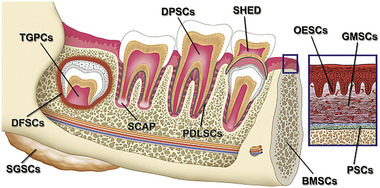
Sources of adult stem cells in the oral and maxillofacial region. BMSCs: bone marrow‐derived MSCs from orofacial bone; DPSCs: dental pulp stem cells; SHED: stem cells from human exfoliated deciduous teeth; PDLSCs: periodontal ligament stem cells; DFSCs: dental follicle stem cells; TGPCs: tooth germ progenitor cells; SCAP: stem cells from the apical papilla; OESCs: oral epithelial progenitor/stem cells; GMSCs: gingiva‐derived MSCs), PSCs: periosteum‐derived stem cells; SGSCs: salivary gland‐derived stem cells. Reproduced with permission.^[^ ^245^ ^]^ Copyright 2012, Elsevier.

Stem cells used with the scaffolds, substrates, and growth factors are important for regenerative dentistry, as in all facets of regenerative medicine. Teeth are an excellent source of stem cells and can be easily obtained by extracted or exfoliation of primary teeth. The stem cells isolated from oral cavity including gingiva, oral mucosa, tooth, and the periodontal ligament are dental pulp stem cells, periodontal ligament stem cells (PDLSC), stem cells from apical papilla, dental follicle stem cells, and gingival mesenchymal stem cells (**Figure** **14**).^[^ ^244^ ^]^

Dental pulp stem cells contained within a hydroxyapatite/silk fibroin scaffold that was decorated with ultra‐small iron oxide differentiated into odontoblast‐like cells. These osteoblast‐like cells retrieved from 8‐week mice expressed dentin sialophosphoprotein and dentin matrix protein 1.^[^ ^246^ ^]^ Another study compared the differences between the use of apical complex cells (ACCs) and dental pulp stem cells for regeneration of the dentin‐pulp complex. The results of this study indicated that the coronal pulp source (i.e., dental pulp stem cells) were more suitable for regeneration because of their more homogenous cell lineage and favorable differentiation potential.^[^ ^247^ ^]^ Granulocyte colony‐stimulating factor, which has stimulating effects on stem cell mobilization, was coupled with dental pulp stem cells and demonstrated potent proliferation and migration potential.^[^ ^248^ ^]^ In another study, the performance of dental pulp stem cells on different types of layer‐by‐layer modified gelatin sponge scaffolds, including gelatin (G), gelatin sponge and polylysine modification (G + P), gelatin sponge and mineralization modification (G + M), and gelatin sponge and mineralization  and poly‐l‐lysine modifications (G + M + P), were evaluated via in vitro and in vivo tests. The results indicate that the G + M + P scaffold exerted the strongest effect on stem cell differentiation.^[^ ^249^ ^]^ Evaluation of the effects of BMP‐7 on differentiation and proliferation of dental pulp stem cells indicated that this growth factor induced odontogenic differentiation via the Smad5 signaling pathway.^[^ ^250^ ^]^ Under the same culture conditions, bone extracellular matrix‐hydrogel scaffolds were found to be more effective for odontogenic differentiation of dental pulp stem cells than collagen scaffolds.^[^ ^251^ ^]^ A clinical study identified that collagen sponges containing dental pulp stem cells enhanced periodontal regeneration after 1 year of treatment. The presence of collagen scaffold in this method supported stem cell proliferation and differentiation during the first week of administration.^[^ ^252^ ^]^

Regenerative endodontic procedure aims to reconstruct dental pulp tissue in the endodontic space of devitalized teeth with three main goals: 1) resolution of clinical signs and symptoms; 2) tooth maturation; and 3) return of neurogenesis.^[^ ^253^ ^]^ Although it is challenging to achieve true regeneration of pulp/dentin complex, major advances have been made in the field of regenerative endodontics. A recent example includes the development of fibrin‐based hydrogel supplemented with clindamycin‐loaded poly‐lactic acid nanoparticles. This approach to regenerative endodontics was shown to have good antibacterial effect against E. faecalis and promising biocompatibility properties since the synthesis of type I collagen was also observed when testing this hydrogel.^[^ ^254^ ^]^ Similarly, excellent results in terms of antibacterial, angiogenic, and odontogenic effects were reported when bioactive glass nanospheres were used for the delivery of ions and growth factors. The release of copper ions and epidermal growth factor from gass nanospheres clearly demonstrated the efficacy of nanotherapy in suppressing the bacterial growth of E. faecalis and simulation of angiogenesis within endothelial cells.^[^ ^255^ ^]^ Last, less complicated cases where only pulp is exposed and where no infection is present can be treated by direct pulp capping with hydroxyapatite nanoparticle powder combined with fibroblast gowth factor.^[^ ^256^ ^]^

PDLSC‐based periodontal treatment has been shown to be effective in reducing inflammation, enhancing bone regeneration, and preventing tooth loss by generating typical cementum/periodontal ligament‐like structures.^[^ ^257^ ^]^ The use of allogeneic PDLSCs induced periodontal tissue regeneration and reduced inflammation in vivo periodontitis models. The use of PDLSCs and human umbilical vein endothelial cells for periodontal regeneration was investigated via the preparation of a 3D cell sheet that was subsequently implanted into immunodeficient mice. The result of that study revealed the potential effectiveness of this new type of system for regenerating the periodontal treatment.^[^ ^258^ ^]^

Stem cells derived from human exfoliated deciduous teeth (SHED) have been shown to be capable of inducing new bone formation in craniofacial bone regeneration.^[^ ^259^ ^]^ These cells possess high proliferative capacity, immunosuppressive ability, and eliminated oncogenesis risks.^[^ ^260^ ^]^ The effect of SHED on bone tissue regeneration was compared with dental pulp stem cells and DPSCs and bone marrow mesenchymal stem cells. The result identified the potential of SHED in bone tissue regeneration. Although all three cell lines showed the same bone formation, the SHED cell line had the most prominent osteoid production and collagen fiber distribution.^[^ ^261^ ^]^

### 7.7 Oral Cancer

One of the most common forms of head and neck cancers is oral cancer. This malignant condition includes cancer of the lip, tongue, mouth, the floor of mouth, and different parts of the oral cavity.^[^ ^262^, ^263^, ^264^ ^]^ Like other forms of cancer, the aetiology of oral cancer has not been completely elucidated, but it is interesting to mention that, in the light of recent findings, a link between oral microbiota and cancer development has been found. Periodontitis, a disease affecting tooth's supporting tissue, increases the possibility for the development of oral cancer. *P. gingivalis* and *F. nucleatum*, common pathogens in periodontitis‐affected patients, have carcinogenic potential due to their ability to influence cell apoptosis, activate cell proliferation, and even produce carcinogens.^[^ ^265^ ^]^ Oral cancer is considered the major challenge in dental public health because it has high mortality rate. There were 3 54 864 oral cancer cases worldwide in 2018 alone, with up to 177.384 deaths.^[^ ^266^ ^]^ Southern Asia and Pacific islands demonstrate high incidence of oral cancer and among them, countries such as India and Sri Lanka claim the highest incidence of oral cancer.^[^ ^266^ ^]^ Based on estimates, the overall 5‐year survival rate of oral cancer patients in the US is 65%, while this number substantially reduces to 27% in advanced oral cancer.^[^ ^267^ ^]^

**Figure 15:**
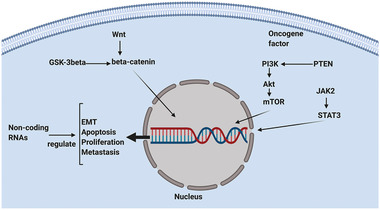
Oral cancer progression is mediated by different molecular pathways. EMT, apoptosis resistance, metastasis, and proliferation are the major features of oral cancer cells. Molecular pathways, as shown in this figure, can act as upstream mediators in their regulation. GSK, glycogen synthase kinase; EMT, epithelial‐to‐mesenchymal transition; PTEN, phosphatase and tensin homolog, JAK2, Janus kinase 2; STAT3, signal transducer and activator of transcription 3; PI3K, phosphatidylinositol 3‐kinase; mTOR, mammalian target of rapamycin, Akt, protein kinase‐B.

Over the recent years, various strategies have been used to improve the overall 5‐year survival rate of oral cancer patients. These strategies include radiotherapy, chemotherapy, and surgery. However, no significant improvement has been witnessed. There are different reasons why currently applied therapies are not completely successful in promoting overall survival of oral cancer patients. This may be attributed to some abnormal features of oral cancer cells that render them distinct from normal cells. Similar to other kinds of cancers, oral cancer cells demonstrate high potential in proliferation and invasion. These aggressive behaviors are responsible for resistance of oral cancer cells toward therapies. A variety of molecular pathways accounting for oral cancer malignancy have been identified. Metastasis of oral cancer cells is mediated via upregulation of epithelial‐to‐mesenchymal transition,^[^ ^268^ ^]^ matrix metalloproteinases,^[^ ^269^ ^]^ microRNAs,^[^ ^270^ ^]^ and long non‐coding RNAs.^[^ ^271^ ^]^ In addition, PI3K/Akt/mTOR,^[^ ^272^ ^]^ Wnt,^[^ ^273^ ^]^ STAT3,^[^ ^274^ ^]^ and TGF‐*β* ^[^ ^275^ ^]^ contribute to oral cancer proliferation. A critical issue is the interaction among the aforementioned pathways, and other signaling networks that participate in oral cancer progression. In addition, oral cancer cells can switch molecular pathways to induce chemoresistance and radio‐resistance.^[^ ^276^, ^277^ ^]^ **Figure** **15** represents a summary of molecular pathways involved in oral cancer progression.

As the underlying molecular pathways and mechanisms involved in oral cancer progression have been identified, therapies have been directed toward their targeting. However, there are issues related to these therapies. For instance, synthetic anti‐tumor agents suffer from adverse reactions, and they cannot accumulate sufficiently in cancer cells due to the presence of drug transporters such as P‐glycoprotein that prevent the entrance of anti‐cancer into cancer cells.^[^ ^278^ ^]^ Phytochemicals with anti‐tumor activity suffer from poor bioavailability.^[^ ^279^ ^]^ Genetic tools including the CRISPR/Cas9 system and siRNA have off‐targeting and may be degraded in the blood circulation, reducing their efficiency in gene silencing. Therefore, there is an urgent need for developing novel strategies in oral cancer therapy.

As s mentioned, each strategy adopted for oral cancer therapy suffers from some drawbacks. These disadvantages may be solved using nanoparticles. Nanoparticles command an important stance in anti‐cancer therapy because they can reduce the adverse effects of anti‐cancer drugs by reducing their dosages and simultaneously maintaining the anti‐cancer properties of those drugs.^[^ ^280^ ^]^ In addition, nanoparticles improve the bioavailability of plant derived‐natural products, prevent siRNA degradation, and provide targeted delivery of CRISPR/Cas9 system. Recently, Fe_3_O_4_ magnetic nanoparticles have been used for delivery of siRNA for treating oral cancer. Bcl‐2 and survivin are upregulated during proliferation of oral cancer. Increase in cellular uptake of siRNA‐Bcl‐2 and siRNA‐survivin occurs with the use of magnetic nanoparticles. This results in enhanced efficacy of gene silencing, which disrupts oral cancer growth and viability.^[^ ^281^ ^]^

It was previously mentioned that oral cancer cells are capable of inducing chemoresistance. MSNs possess the capacity of encapsulating siRNA‐MDR1 and TH287 in interfering with proliferation of oral cancer cells and suppressing chemoresistance. Of note, the capacity of MSNs in selective targeting of oral cancer cells may be enhanced via surface modification. CD44 receptors are overexpressed on the surface of oral cancer cells. Hyaluronic acid modification of MSNs promotes its capacity in targeting oral cancer cells with CD44‐overexpression. This increases cellular uptake of the functionalized MSNs.^[^ ^282^ ^]^ Wnt activation is correlated with cancer metastasis via induction of epithelial‐to‐mesenchymal transition. Hence, down‐regulation of Wnt signaling is important in inhibiting cancer metastasis. Polyethylene glycol‐polyethyleneimine‐chlorin e6 (PEG‐PEI‐Ce6) nanoparticles have been designed for delivery of siRNA‐Wnt1 in oral cancer therapy. Exposing oral cancer cells (KB cells) to PEG‐PEI‐Ce6 nanoparticles containing siRNA‐Wnt1 resulted in inhibiting nuclear translocation of *β*‐catenin. This, in turn, suppressed hat is in favor of suppressing epithelial‐to‐mesenchymal transition and metastasis via vimentin down‐regulation. Furthermore, these nanoparticles promote the efficacy of siRNA in silencing Wnt1.^[^ ^283^ ^]^ These studies demonstrate the potential role of nanoparticles in delivery of siRNA for oral cancer therapy. To date, there is no study evaluating role of nanoparticles for delivery of CRISPR/Cas9 in oral cancer therapy. Further studies have to be focused on this subject.

Apart from gene delivery, nanoparticles may be used for the delivery of anti‐cancer drugs. As previously mentioned, chemoresistance is an increasing challenge for effective treatment of oral cancer. Chemotherapeutic agents possess side effects, urging scientists to apply low levels of these agents. Consequently, attention has been directed toward the use of nanoparticles for delivery of anti‐cancer drugs. Recently, catechol‐modified chitosan/hyaluronic acid nanoparticles (Cat‐NPs) have been designed for delivery of doxorubicin. Catechol modification of nanoparticles promotes its mucoadhesive properties that are beneficial for local delivery. The Cat‐NPs release doxorubicin in a prolonged‐release manner and significantly reduce the half maximal inhibitory concentration (IC_50_) value of doxorubicin. Enhanced accumulation of doxorubicin occurs following the use of Cat‐NPs. Oral cancer cells undergo apoptosis upon exposure to Cat‐NPs.^[^ ^284^ ^]^ The same features occur with the use of cisplatin. Nanoparticles augment the efficacy of cisplatin in apoptosis induction of oral cancer cells due to increased internalization.^[^ ^285^ ^]^ Nanoparticles not only enhance intracellular accumulation of chemotherapeutic agents. They also provide a platform for co‐delivery of other anti‐cancer agents. For example, curcumin is a phytochemical derived from rhizome of *Curcuma longa*. Curcumin possesses potent high anti‐cancer activity against oral cancer cells. This plant derived‐natural product can suppress epithelial‐to‐mesenchymal transition and proliferation (apoptosis) via affecting molecular pathways such as c‐Met and ERK.^[^ ^286^, ^287^ ^]^ Nanoemulsions have been used for co‐delivery of curcumin and 5‐fluorouracil. The release of these anti‐cancer agents occurs at 4 days with higher release in acidic pH, similar to the tumor microenvironment. By upregulating p53, p21, and Bax, and down‐regulating Bcl‐2, curcumin sensitizes oral cancer cells to 5‐fluorouracil chemotherapy. Nanoemulsions provide targeted delivery and sustained‐release that are of importance in promoting anti‐cancer activity of both curcumin and 5‐fluorouracil.^[^ ^288^ ^]^ These studies are in agreement with the capability of nanoparticles as synergistic factors in anti‐cancer therapy. **Table** **2** summarizes the recent progress in using nanoaprticles for therapy against oral cancer.

**Table 2:** Representative examples of the use of nanovehicles as agents for oral cancer therapy

Nano‐vehicle	Nature of study	Cell line/animal model	Particle size [nm]	Zeta potential [mV]	Encapsulation efficacy [%]	Remarks	Ref.
Hydrogel	In vitro In vivo	HSC‐3 and UM1 cells OSCC xenograft	20–30	‐	‐	Isoguanosine‐borate‐guanosine hydrogels have high stability and biocompatibility. Suppress tumor growth	^[^ ^289^ ^]^
Polymeric copolymers	In vitro	KB human epithelial carcinoma and YD‐38 squamous carcinoma cells	96.2–189.1	‐	11.8–28.3	High cellular uptake. Redox responsive. Surface modification with folate receptor in promoting their internalization. Enhance generation of reactive oxygen species. High cytotoxicity	^[^ ^290^ ^]^
Chitosan nanoparticle	In vitro Ex vivo	SCC‐9 cells	188	44.8	89.1	Initial burst release followed by sustained release. Enhance drug penetration by three times. Trigger cancer cell death.	^[^ ^291^ ^]^
PLGA nanoparticles	In vitro	SCC‐9 cells	150	‐	‐	Prolonged release with 60% release in 9 days. Reduce cell viability (>50%)	^[^ ^292^ ^]^
N‐doped carbon quantum dot	In vitro	FaDu and HaCaT cells	48	−50	‐	Simultaneous imaging and photothermal therapy. High internalization.	^[^ ^293^ ^]^

### 7.8 Oral Mucositis

Oral mucositis is one of the most common side effects during head and neck cancer treatment.^[^ ^294^ ^]^ It is observed in 80% of patients undergoing radiotherapy and 100% of patients receiving high‐dose chemotherapy.^[^ ^295^ ^]^ It is associated with pain and discomfort, susceptibility to bacterial and fungal infections which can lead to sepsis in most severe cases. To prevent possible complications, 4% rebamipide solution is usually prescribed to patients undergoing head and neck cancer therapy. However, its greatest flaw is the transient presence in the oral cavity and, consequently, reduced drug effectiveness. To overcome this obstacle, chitosan‐coated poly(lactide*‐co‐*glycolide) nanoparticles have been synthesized as carriers for rebamipide. The mean volume diameter of these nanoparticles was ≈100 nm with positive surface charge being one of their major characteristics. Coating the nanoparticles with chitosan enabled them to be retained in the oral cavity for more than 6 h due to their electrostatic interaction with the negatively‐charged carboxyl groups of mucin within the oral mucosa. The rebamipide chitosan‐coated nanoparticles demonstrated superior results with shortened treatment time when compared to rebamipide suspension alone.^[^ ^296^ ^]^ Similarly, promising results in treating induced oral mucositis in hamsters were achieved using 250 µg kg^−1^ gold nanoparticles.^[^ ^297^ ^]^

### 7.9 Oral Medicine

Oral ulcers (commonly known as canker sore or recurrent aphthous stomatitis) are generally painful lesions that affect oral cavity and have various etiologic factors. They are often associated with infections, immune disorders, trauma, or neoplasms.^[^ ^298^ ^]^ The treatment of oral ulcers can be challenging and depends on the etiologic factor, but in most cases consists of reducing or eliminating the pain and preventing secondary infections. Promising results in healing of radiation‐induced oral ulcers in mice have been obtained by applying a combination of Aloe vera and silver nanoparticles on the affected sites.^[^ ^299^ ^]^ In clinical trials involving human subjects, a new therapeutic approach, that lies within the range of nanoparticles, was introduced by applying oromuco‐adhesive films containing propolis extract. This drug delivery system was able to prolong the duration of pain relief and enhance the healing processes.^[^ ^300^ ^]^

## 8 Conclusion and Outlook

As the reader may have already perceived, the use of micro and nanosystems as drug delivery carriers is readily expanding in dentistry and the oral health sector. In the future, more and more products will be approved and be available on the market. Nanoparticles present exceptional features such as a tunable structure and intelligent properties such as bio‐adhesive behavior or stimuli‐responsive ability. These properties may be harnessed during drug formulation, particularly for treating diseases such as antiviral therapy in which relatively few treatment techniques are available. This is for sure one of the main challenges addressed by today's world of scientific research in this field. The development of smart nanomaterials represents a pivotal point for many applications and various treatments such as gene and peptide delivery; because the exceptional improvements can be obtained working with precise and timely drug release. Internal or external functionalization, the tuning of the core formulation or the conjugation of the nanostructures with specific molecules enable to impose to the whole systems specific chemical or physical properties. In this direction, they represent good examples of effective strategies to obtain precise features in terms of drug release properties for these impending technologies.

Oral cancer possesses high morbidity and mortality. A large number of new cases are diagnosed with oral cancer annually. It is beneficial to provide effective treatment. Conventional therapies are no longer effective in oral cancer treatment because of the side effects of anti‐cancer drugs and the emergence of a new phenomenon known as chemoresistance. Nanoparticles can promote the anti‐tumor activity of chemotherapeutic agents via providing targeted delivery and enhancement of cellular uptake. The adverse effects of chemotherapeutic agents are reduced substantially upon the use of nanoparticles due to loading of low concentrations of the respective drug. As gene therapy is beneficial in suppressing oral cancer proliferation and metastasis, and improving overall 5‐year survival rate of oral cancer patients, genetic tools can be encapsulated in nanoparticles to protect them against degradation, elevate their internalization and enhance their efficiency in gene silencing. The potential of nanoparticles in targeted delivery can be boosted using surface modification, for example, by the use of hydroxyapatite. Such benefits have made nanoparticles an inevitable part of oral cancer therapy.

The increasing number of scientific papers that report the synthesis of different nanoparticles and their application in the dental field is a clear indicator of how promising the obtained results are. In the future, it would be of great interest to conduct clinical trials that would confirm the beneficial effect that nanoparticles provide in treating pathological conditions affecting the oral cavity. Beyond the advantages listed above some challenges must be solved in order to make feasible the translation to the clinic and subsequent commercialization. First, deeper investigations should be done on possible toxic effects of nanoparticles in order to improve their biocompatibility. Because of this many preclinical studies are needed, investigating the immune system interactions and unanticipated toxicities. Second, their target activity is a pivotal point, and improving the specificity of functional nanoparticles‐based formulation is essential. Then, the preservation of the pharmacological activity of nanoparticles when binding with target should be maintained. In this framework, nanodrug structure design and fabrication protocol are essential, considering that many biological mechanisms related to nanoparticle effects on the human body are still largely unknown and because of this clinical efficacy studies are required. Last, the facility of scale‐up production, the control over critical design features, and the final cost, are also extremely important and they make a big difference between dental applications and other diseases where the cost factor is less important.

## Conflict of Interest

The authors declare no conflict of interest.
